# Ionic Liquid-Based Strategy for Predicting Protein Aggregation Propensity and Thermodynamic Stability

**DOI:** 10.1021/jacsau.2c00356

**Published:** 2022-09-09

**Authors:** Talia A. Shmool, Laura K. Martin, Richard P. Matthews, Jason P. Hallett

**Affiliations:** † Department of Chemical Engineering, Imperial College London, South Kensington Campus, London SW7 2AZ, U.K.; ‡ Department of Engineering Science, University of Oxford, Parks Road, Oxford OX1 3PJ, U.K.

**Keywords:** ionic liquids, therapeutic aggregation, thermodynamic stability, storage stability, predictive strategy

## Abstract

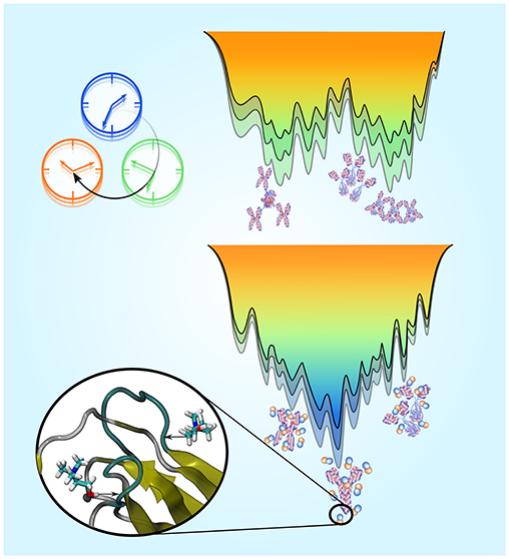

Novel drug candidates are continuously being developed to combat the most life-threatening diseases; however, many promising protein therapeutics are dropped from the pipeline. During biological and industrial processes, protein therapeutics are exposed to various stresses such as fluctuations in temperature, solvent pH, and ionic strength. These can lead to enhanced protein aggregation propensity, one of the greatest challenges in drug development. Recently, ionic liquids (ILs), in particular, biocompatible choline chloride ([Cho]Cl)-based ILs, have been used to hinder stress-induced protein conformational changes. Herein, we develop an IL-based strategy to predict protein aggregation propensity and thermodynamic stability. We examine three key variables influencing protein misfolding: pH, ionic strength, and temperature. Using dynamic light scattering, zeta potential, and variable temperature circular dichroism measurements, we systematically evaluate the structural, thermal, and thermodynamic stability of fresh immunoglobin G4 (IgG4) antibody in water and 10, 30, and 50 wt % [Cho]Cl. Additionally, we conduct molecular dynamics simulations to examine IgG4 aggregation propensity in each system and the relative favorability of different [Cho]Cl-IgG4 packing interactions. We re-evaluate each system following 365 days of storage at 4 °C and demonstrate how to predict the thermodynamic properties and protein aggregation propensity over extended storage, even under stress conditions. We find that increasing [Cho]Cl concentration reduced IgG4 aggregation propensity both fresh and following 365 days of storage and demonstrate the potential of using our predictive IL-based strategy and formulations to radically increase protein stability and storage.

## Introduction

A staggering figure of over 96% of drugs fail during development.^1^ The abandoned therapeutics, long development timelines, and high costs incurred are major roadblocks impacting industry and patients.^1,2^ Currently, monoclonal antibodies dominate the pharmaceutical sector, offering promising treatment options for life-threatening diseases.^2^ However, antibodies are inherently prone to form aggregates in solution and thus are typically stored under −20 °C to prolong shelf life.^3^ To evaluate and predict therapeutic shelf life and determine the candidates of reduced aggregation propensity that should advance to clinical trials, phenomenological and extensive empirical experimental screening is conducted.^1,4,5^ However, existing approaches are of high cost, time consuming, and challenging in terms of accuracy.^5−8^ Thus, novel strategies for predicting protein aggregation propensity and identifying stabilizing solutions in formulation development are required.

Major factors shown to affect protein aggregation behavior in solution include temperature, pH, salt concentrations, solution ionic strength, and exposure of solvent-accessible regions in the protein.^9,10^ Generally, shifting the pH toward the isoelectric point and raising solution ionic strength can disturb the electrostatic interactions between the protein surfaces and the solvent and protein, required for protein hydration.^4,8,11,12^ Additionally, solubility is reduced as attractive intermolecular forces are increased, leading to precipitation and enhanced aggregation propensity.^3,8,12^ Furthermore, even slight temperature fluctuations can result in protein aggregation, particularly over long term-storage.^2,4,5,8^ Thus, when developing a protein therapeutic formulation, to enhance solubility while preventing aggregation, as a first stage, solution pH is adjusted and solutions with minimal salt concentration and low ionic strength are typically used.^2,3,13^

Recently, we developed an energy-guided experimental methodology to determine the thermodynamic properties and enhance the conformational stability of biomolecules using ionic liquids (ILs).^14,15^ ILs are composed entirely of ions, exist in the liquid state below 100 °C, and are of high chemical tunability.^16−18^ Uniquely, ILs can be tactically used to enhance the structural and thermal stability of diverse proteins.^14−18^ While the molecular mechanisms remain largely mysterious, enhanced thermodynamic stability of the native protein state in IL systems has been rationalized in terms of hydrophobic confinement, specific ion effects, and the Hofmeister series.^15,18,19^ We have shown an increase in the structural and thermal stability of therapeutics in ILs, attributed to protein sequestration, energetically favorable from both an entropic and enthalpic perspective.^14,15^ Notably, the formation of thermally and structurally stable protein structures, following synthesis, is a thermodynamically controlled process.^19^ We consider that by determining the thermodynamic properties, including the transition enthalpies, entropies, and free energies, for a given protein therapeutic in IL, it will be possible to assess and predict the aggregation propensity and thermal and structural stability of the therapeutic. Moreover, we can provide key insight into the molecular mechanisms of protein aggregation suppression by ILs. Potentially, by harnessing the unique properties of ILs, we can extend the shelf-life of pharmaceuticals and revive lost therapeutic candidates.

Herein, we mimic industrial storage conditions and conduct a systematic study to develop an IL-based strategy for predicting thermodynamic stability and protein aggregation as well as product shelf life. We select immunoglobin G4 (IgG4) of inherently high aggregation propensity^3^ as our model protein and choline chloride ([Cho]Cl) as our IL-based solvent system. [Cho]Cl was chosen as it is biocompatible, of low molecular weight, with high solubility in water, and can be easily synthesized with high purity and low cost.^16,20^ Additionally, [Cho]Cl is a simple organic compound, ideal for the development of a novel robust predictive approach for biotherapeutics. Previously, we found that the optimal proportions of 10, 30, and 50 wt % of [Cho]-based ILs significantly enhanced the conformational stability of native and modified proteins in water.^15^ In light of these findings, we prepare a series of formulations including IgG4 in 10, 30, and 50 wt % [Cho]Cl and IgG4 in water control. While we account for the dilution factor of injectable therapeutics, our formulations contain [Cho]Cl at relatively higher proportions compared to the concentrations of excipient salts in therapeutic formulation.^13^ Nonetheless, traditional salts, such as sodium chloride, at similar concentrations to the [Cho]Cl content used herein, have also been shown to enhance the solubility and structural stability of globular proteins and injectable drug formulations.^21,22^ In the absence of traditional protective agents, we investigate the effects of pH, ionic strength, and temperature on IgG4 structural and thermal stability and aggregation propensity, as fluctuations in these conditions during industrial and biological processes can enhance aggregation behavior.^3,4,8^ We perform pH measurements and monitor IgG4 surface charge using zeta potential measurements and conduct dynamic light scattering (DLS) measurements to examine the effect of pH and ionic strength on IgG4 aggregation propensity. We acknowledge that it would be more straightforward to individually examine the influence of pH on protein surface charge and that of varying ionic strength on aggregation propensity; however, singular factors are not isolated in biological, extracellular environments. Thus, we purposefully combine and naturally tune the pH and ionic strength and systematically examine the united influence of these factors on IgG4 surface charge and aggregation propensity. As such, we create realistic scenarios, triggering protein misfolding, to provide information and develop a strategy that can be more readily translated to preselection and optimization of pharmaceutical candidates. Furthermore, to develop an improved accelerated stability testing approach, we conduct temperature variable circular dichroism (CD) experiments to investigate temperature-induced conformational changes and the folding pathways of IgG4 in each system. We perform molecular dynamics (MD) simulations at both 25 and 97 °C, aiming to connect our experimental observations and gain further insight into IgG4 dynamics and aggregation propensity in each system. Based on our initial findings of the structural, thermal, and thermodynamic stability of IgG4 in each system, we predict the aggregation propensity and thermodynamic properties of each IgG4 formulation following 365 days of storage under 4 °C. To test the validity of our initial predictions, we reassess each system by all measurements following storage. It is worth noting that while several studies have examined the storage stability of globular proteins and enzymes in IL,^23−25^ to our knowledge, no study to date has examined the stability of antibodies in choline-based ILs over such an extensive period.

## Results

### Aggregation Propensity of Fresh IgG4 in Water and [Cho]Cl Solutions of Varying pH and Ionic Strength

By pH measurements, we found that by increasing [Cho]Cl concentration, with no buffer adjustment, we could naturally lower the pH from 6.35 in water to 5.86, 5.73, and 5.47 in 10, 30, and 50 wt % [Cho]Cl, respectively, and increase ionic strength (0.760, 2.15, and 3.58 M for 10, 30, and 50 wt %, respectively) (Table S1). In agreement with the literature, we found that the mean surface charge increased with rising ionic strength.^26,27^ Specifically, we observed an increase from 6.2 ± 0.6 to 9.5 ± 0.9 mV for 10–50 wt %, respectively, lowest for IgG4 in water (2.3 ± 0.8 mV). This is also consistent with previous work showing that protein surface charge increases in solutions of higher ionic strength and lower pH.^28−32^

Notably, the rise in ionic strength and lower pH of the 50 wt % [Cho]Cl environment can increase protein surface charge and promote misfolding and precipitation.^17,29,30^ However, our DLS measurements showed that in 50 wt % [Cho]Cl, the mean hydrodynamic diameter (*D*_h_) of fresh IgG4 was the lowest (135 ± 4 nm) and the highest for water (410 ± 10 nm), closest to physiological pH (Table S1). We also observed a relatively lower PDI for all [Cho]Cl solutions compared to water. It should be noted that our findings are in agreement with previous work in that greater aggregation propensity should be observed close to the isoelectric point of the protein,^5,10,27,28^ in this case, IgG4 in water. Overall, these results highlight the inherently high aggregation propensity of fresh IgG4, lowered with [Cho]Cl addition.

We predicted that if the behavior of IgG4 is strongly controlled by [Cho]Cl, following 365 days of storage under 4 °C, we would observe the same rank order of reduced aggregation propensity and increase in zeta potential values with greater [Cho]Cl concentration. Accordingly, we also predicted minimal pH fluctuation for all [Cho]Cl solutions. These predictions relied on the assumption that IgG4 aggregation is hindered by [Cho]Cl addition and the ions of [Cho]Cl form low molecular weight liquids that remain stable over extended storage.

### Validating Initial Predictions of IgG4 Physical Properties and Aggregation Propensity

**Figure 1:**
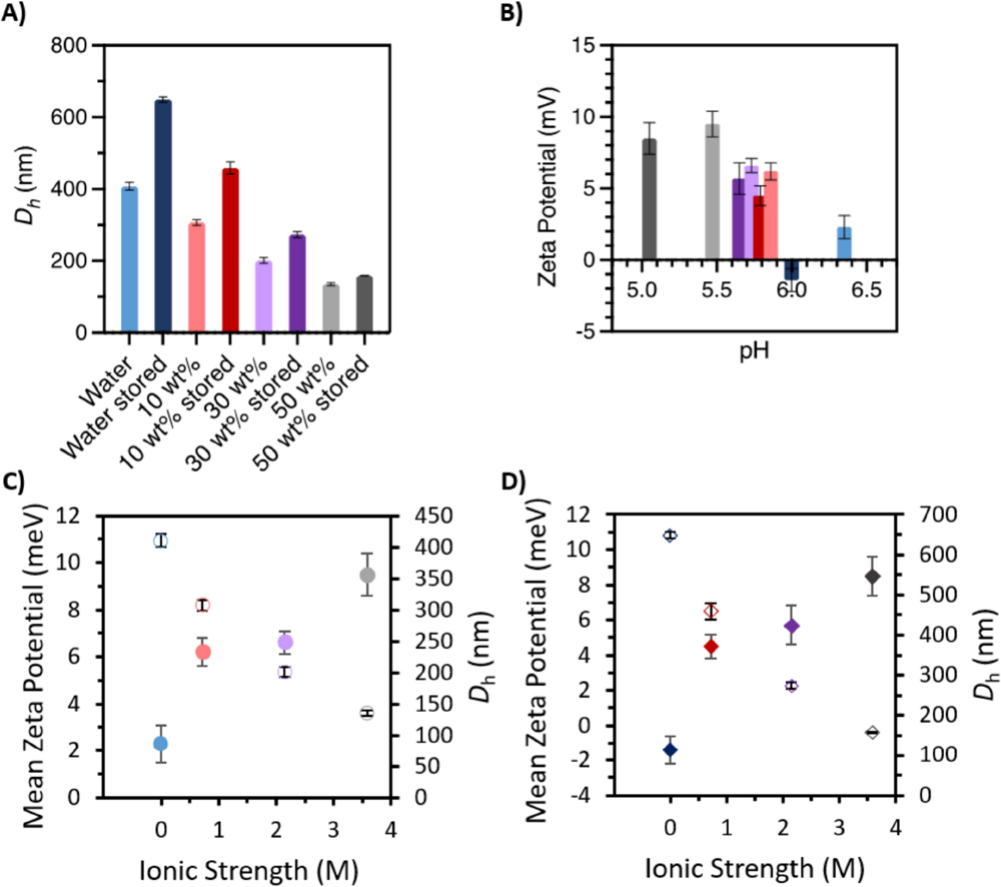
IgG4 in water (blue), 10 wt % [Cho]Cl (red), 30 wt % [Cho]Cl (purple), and 50 wt % [Cho]Cl (gray) with light and dark shades showing fresh and stored samples, respectively. (A) Mean hydrodynamic diameter (*D*_h_); (B) pH versus mean zeta potential for fresh and stored samples ± SD (*n* = 3). Ionic strength versus mean surface charge (solid circles) and hydrodynamic diameter (unfilled circles) for (C) fresh and (D) stored samples ± SD (*n* = 3). See the Supporting Information for values (Table S1) and significance tests.

Following 365 days of storage at 4 °C, pH, DLS and zeta potential measurements confirmed our initial predictions (observations overlayed for emphasis in Figure 1A, B). For IgG4 stored in [Cho]Cl, the pH of each solution only slightly decreased following storage (5.79, 5.65, and 5.05 for stored 10, 30, and 50 wt % [Cho]Cl, respectively), reflecting the integrity of the [Cho]Cl solutions and ability to maintain the appropriate pH over long-term storage. As we observed for the fresh samples (Figure 1C), the mean zeta potential values for the stored samples increased with rising ionic strength (from 4.5 ± 0.7 to 8.5 ± 1.1 mV for 10–50 wt %, respectively) and decreasing pH (Figure 1B,D). It is worth noting that upon addition of salt, native aggregation can increase,^4,8,30−34^ yet we found the smallest increase in *D*_h_ for stored 50 wt % [Cho]Cl (159 ± 1 nm) followed by 30 and 10 wt % (274 ± 8 and 460 ± 20 nm, respectively) and greatest for water (649 ± 8 nm). Thus, our findings demonstrate that by using a higher [Cho]Cl concentration, intrinsic IgG4 aggregation propensity can be decreased and sustained over long-term storage. We highlight that the consistency in trends following extended storage provides the first piece of evidence that upon exposing an antibody to an IL-based solvent, it is possible to identify, control, and predict the long-term physical properties and stability of aggregate-prone protein systems.

### Ranking Fresh IgG4 Systems by Structural Stability and Identifying the Lowest Energy State

**Figure 2:**
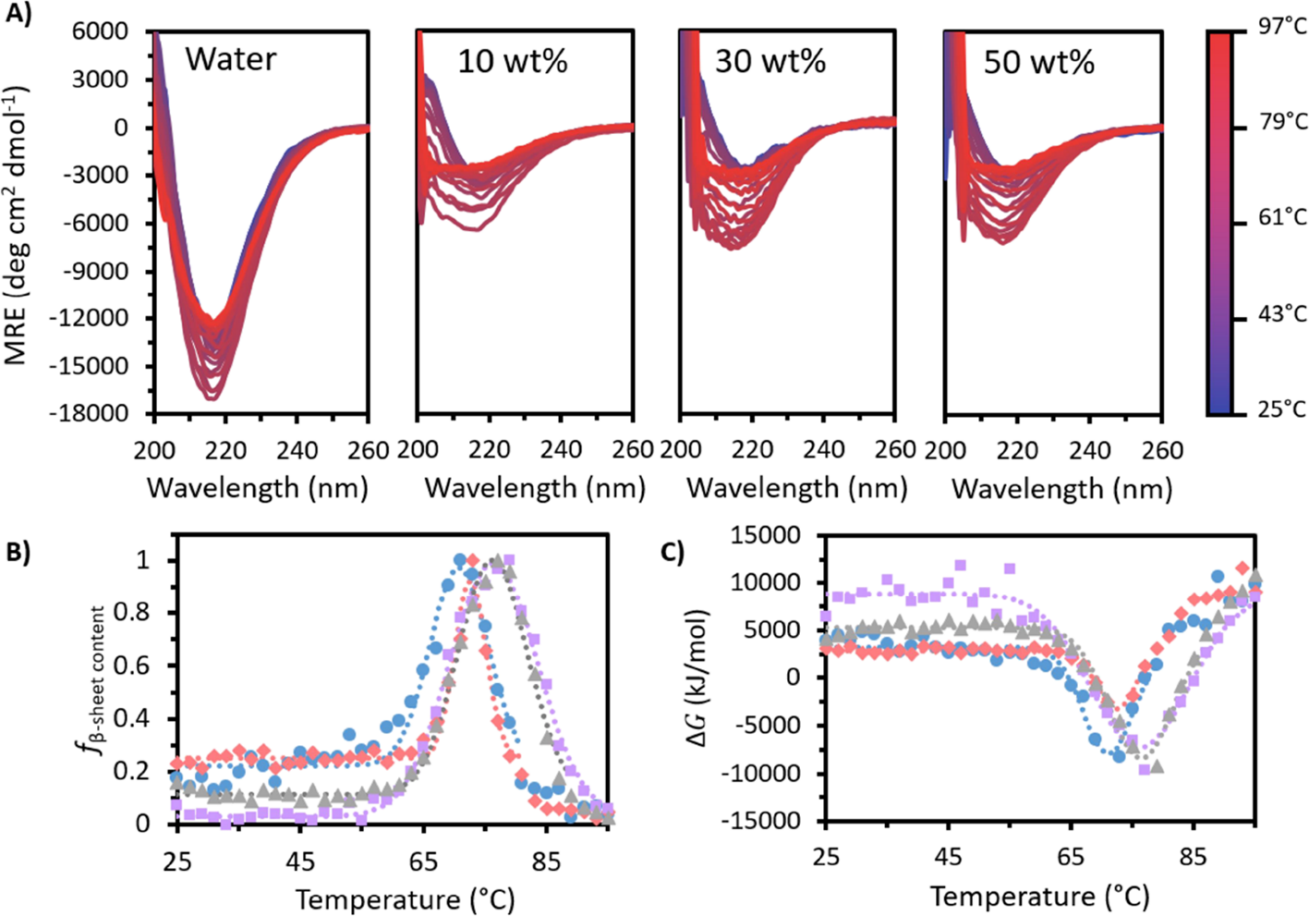
(A) MRE plots for fresh IgG4 in water and 10, 30, and 50 wt % [Cho]Cl. (B) *f*_f_β – sheet content__ and (C) Gibbs free energy (with temperature for fresh water (blue circles), 10 wt % (red diamonds), 30 wt % (purple squares), and 50 wt % [Cho]Cl (gray triangles). Dotted lines show Gaussian curves fitted to each fresh sample data set.

We develop our predictive IL-based strategy and examine heat induced protein aggregation using temperature variable CD experiments. Figure 2A shows that in all cases, fresh IgG4 exhibited a negative band at approximately 218 nm attributed to the β-sheet secondary structure.^14^ In water, IgG4 gave a much deeper initial minimum at 218 nm (−12,800 deg cm^2^ dmol^–1^), with all [Cho]Cl samples showing similar spectra at 25 °C (between −2800 and −3200 deg cm^2^ dmol^–1^). As the temperature was raised, this minimum at 218 nm initially increased in depth and breadth for all samples. Notably, compared to the [Cho]Cl systems, fresh IgG4 in water showed a relatively small change in the mean residue ellipticity (MRE) with temperature due to the very deep initial value. In all cases, the absolute change in MRE was between −3000 to −4500 deg cm^2^ dmol^–1^ (Table S2). As Figure 2A provides a qualitative picture for the structural changes and aggregation behavior of each fresh sample, the MRE data was further analyzed at 218 nm to monitor the relative fractional change in the β-sheet content (*f*_β – sheet content_) (Figure 2B, Table S3). For each fresh system, we observed two distinct sigmoidal regions corresponding to the thermally induced conformational transitions and IgG4 conformational changes.^14,15^ Additionally, we observed that IgG4 in water deviated from Gaussian behavior above 85 °C, showing multiple conformational transitions and lower thermal and structural stability compared to all [Cho]Cl systems (Figure S1). Notably, relative to water, we observed that both the initiation and maximum of the peak in *f*_β – sheet content_ occurred at higher temperatures for the [Cho]Cl systems. This demonstrates the rise in conformational, structural, and thermal stability of fresh IgG4 with increasing [Cho]Cl concentration, similar for 30 and 50 wt % and lower for 10 wt % and water.

Based on our previous work, choline-based ILs can yield well-formed IgG4 secondary structures and structural motifs that are energetically favorable, directing the positioning of the secondary structural elements of IgG4 in space.^14,15,34^ To investigate this in detail, we traced the change in Gibbs free energy for fresh samples (Δ*G*_f_) with temperature. We found that for fresh IgG4 in water, the initial decline began at 57 °C, decreasing to a minimum at 71 °C and then increasing beyond this to 95 °C (Figure 2C). We also observed that fresh water deviated from Gaussian behavior above 85 °C, alike the *f*_β – sheet content_, and yielded a higher RSS value for the obtained fit compared to the fresh [Cho]Cl systems (Tables S4 and S5). Similar to Figure 2B, we found that the negative peak in the plot of Δ*G*_f_ began and reached its minimum value at higher temperatures for the [Cho]Cl samples compared to water. Specifically, for all fresh [Cho]Cl samples, Δ*G*_f_ can be described by a stable period at low and moderate temperatures (from 25 °C to at least 55 °C), followed by a decrease to a minimum then increase to at least the initial value at 97 °C. In fresh 10 wt %, the negative peak in Δ*G*_f_ began at 63 °C, with a minimum reached at 72 °C, while in fresh 30 and 50 wt % [Cho]Cl, the decrease began at 59 and 61 °C, respectively, and both reached a minimum at 76 °C. This demonstrates the increase in the thermodynamic stability of fresh IgG4 at higher [Cho]Cl concentrations and the temperature dependence of Δ*G* and the *f*_β – sheet content_ for each fresh sample.

To further investigate the systems most likely to improve thermodynamic stability, we calculated Δ*G*_min_, as the minimum value on the Gaussian fit of the raw data. Δ*G*_min_ corresponds to the temperature at which the conformational states show the greatest *f*_β – sheet content_. We found that for the fresh [Cho]Cl samples, Δ*G*_f_min__ became more negative with increasing [Cho]Cl content (from −3.3 ± 0.4 kJ/mol at 10 wt % to −7 ± 1 kJ/mol at 30 wt % to −8.3 ± 0.9 kJ/mol for 50 wt %), showing greater thermodynamic stability.^14,15^ In water, Δ*G*_f_min__ was also more negative (−8 ± 1 kJ/mol), but the observed deviation from the Gaussian model at higher temperatures indicated that IgG4 in water is highly aggregation-prone, more so with increasing temperature. Notably, fresh IgG4 in [Cho]Cl systems did not show such deviation from the Gaussian model.

Based on the results for the fresh samples, we expected that following 365 days at 4 °C, IgG4 stored in water and 10 wt % [Cho]Cl would exhibit greater structural changes than 30 and 50 wt %. Given that fresh water showed a deviation from the Gaussian model at higher temperatures, we predicted that stored water would also show this above 80 °C. Moreover, since fresh 10 wt % showed the least negative Δ*G*_f_min__, we also anticipated a distortion from the Gaussian model at higher temperatures for stored IgG4 in 10 wt %. Finally, we expected that compared to Δ*G*_f_min__, for stored water and 10 wt %, Δ*G*_s_min__ would become more negative and change more in magnitude compared to stored 30 and 50 wt % [Cho]Cl. These predictions would hold true if in water and 10 wt % IgG4 aggregation propensity increases over storage, and thus, the Δ*G*_s_min__ values become more negative to compensate and enhance IgG4 thermodynamic stability.^35−38^ Notably, our predictions are made in the framework that the fitted Δ*G* curve essentially demonstrates the force of folding, and Δ*G*_f_min__ can be used to predict Δ*G*_s_min__ and the stored final IgG4 conformations.

### Verifying Predictions and Examining the Final Stored IgG4 Structures

**Figure 3:**
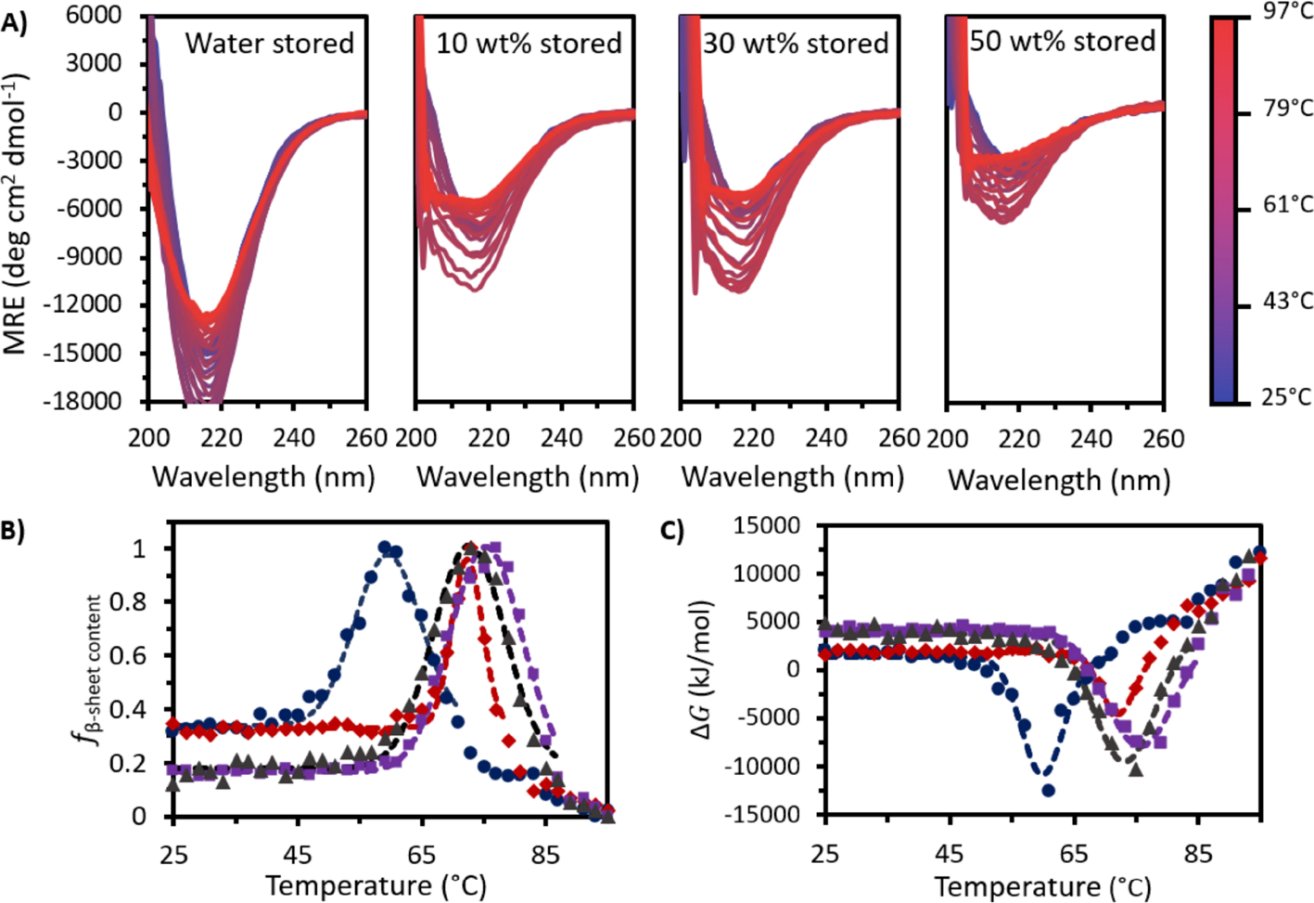
(A) MRE plots following 365 days at 4 °C for stored IgG4 in water and 10, 30, and 50 wt % [Cho]Cl. (B) *f*_s_β – sheet content__ and (C) Δ*G* with temperature for stored water (dark blue circles), 10 wt % (dark red diamonds), 30 wt % (dark purple squares), and 50 wt % (dark gray triangles). Dotted lines show Gaussian curves fitted to each stored sample data set.

Following 365 days at 4 °C, all stored samples showed a loss in secondary structure beyond the temperature at which the negative band reached its maximum amplitude (Figure 3A). This was most pronounced for stored water. We observed a small difference in secondary structure loss for the stored [Cho]Cl samples, in line with our initial projection. Specifically, the well at 218 nm became deeper for all stored samples except for 50 wt % [Cho]Cl, which retained almost an identical initial well depth of −3000 deg cm^2^ dmol^–1^ after storage, compared to −3200 deg cm^2^ dmol^–1^ fresh (Table S2). Furthermore, the increase in well depth with heating was also raised for all stored samples except 50 wt %, indicating a lower loss in secondary structure and reduced IgG4 aggregation propensity with [Cho]Cl addition.

Consistently, each stored sample displayed a Gaussian curve for the *f*_s_β – sheet content__ with temperature (Figure 3B). The peak in *f*_s_β – sheet content__ in water occurred at the lowest temperature, while the stored [Cho]Cl samples exhibited peaks in similar positions. We also found a third sigmoidal region between 79 and 97 °C for stored water and between 81 and 97 °C for stored 10 wt %, satisfying our prediction of deviation from the Gaussian model at higher temperatures for both systems. This indicates greater structural changes for water and 10 wt % compared to 30 and 50 wt % [Cho]Cl. While for each case a different conformational landscape is sampled by IgG4 and β-sheet-rich conformations emerged, no distinct transition above 70 °C was visible for stored IgG4 in 30 and 50 wt %, in line with our prediction of reduced aggregation propensity with increasing [Cho]Cl concentrations.

Overall, for all stored samples, the curve shape for Δ*G*_s_ with temperature (Figure 3C) showed minimal differences to that of Δ*G*_f_ and could be fitted to a Gaussian model. As temperature was raised, Δ*G*_s_ decreased to a minimum, Δ*G*_s_min__, and subsequently increased to greater than its initial value. In the stored [Cho]Cl samples, the Δ*G*_s_ well started at nearly the same temperature as in the fresh samples (65, 61, and 59 °C for 10, 30, and 50 wt %, respectively); and Δ*G*_s_min__ also occurred at very similar temperatures (72, 76, and 73 °C for 10, 30, and 50 wt % [Cho]Cl, respectively). After storage in water, Δ*G*_s_min__ decreased by 11 °C, with the negative peak in Δ*G*_s_ initiating at a much lower temperature than stored [Cho]Cl samples and fresh water, starting at just 45 °C, reaching the minimum at 60 °C and returning to initial values by 71 °C before increasing further. Notably, as predicted, we observed more negative values for Δ*G*_s_min__ for all stored samples compared to fresh Δ*G*_f_min__. Specifically, both stored water (−11 ± 1 kJ/mol) and 10 wt % (−4.6 ± 0.4 kJ/mol) showed the greatest percentage changes (37 and 40% compared to Δ*G*_f_min__, respectively), whereas Δ*G*_s_min__ for stored 30 wt % (−7.9 ± 0.6 kJ/mol) and 50 wt % (−9.5 ± 0.9 kJ/mol) showed smaller relative changes (9 and 14% compared to Δ*G*_f_min__, respectively). This further supports the claim that in higher [Cho]Cl concentrations, the thermodynamic stability of IgG4 is greater and aggregation propensity is reduced.^14,15,37,38^

**Figure 4:**
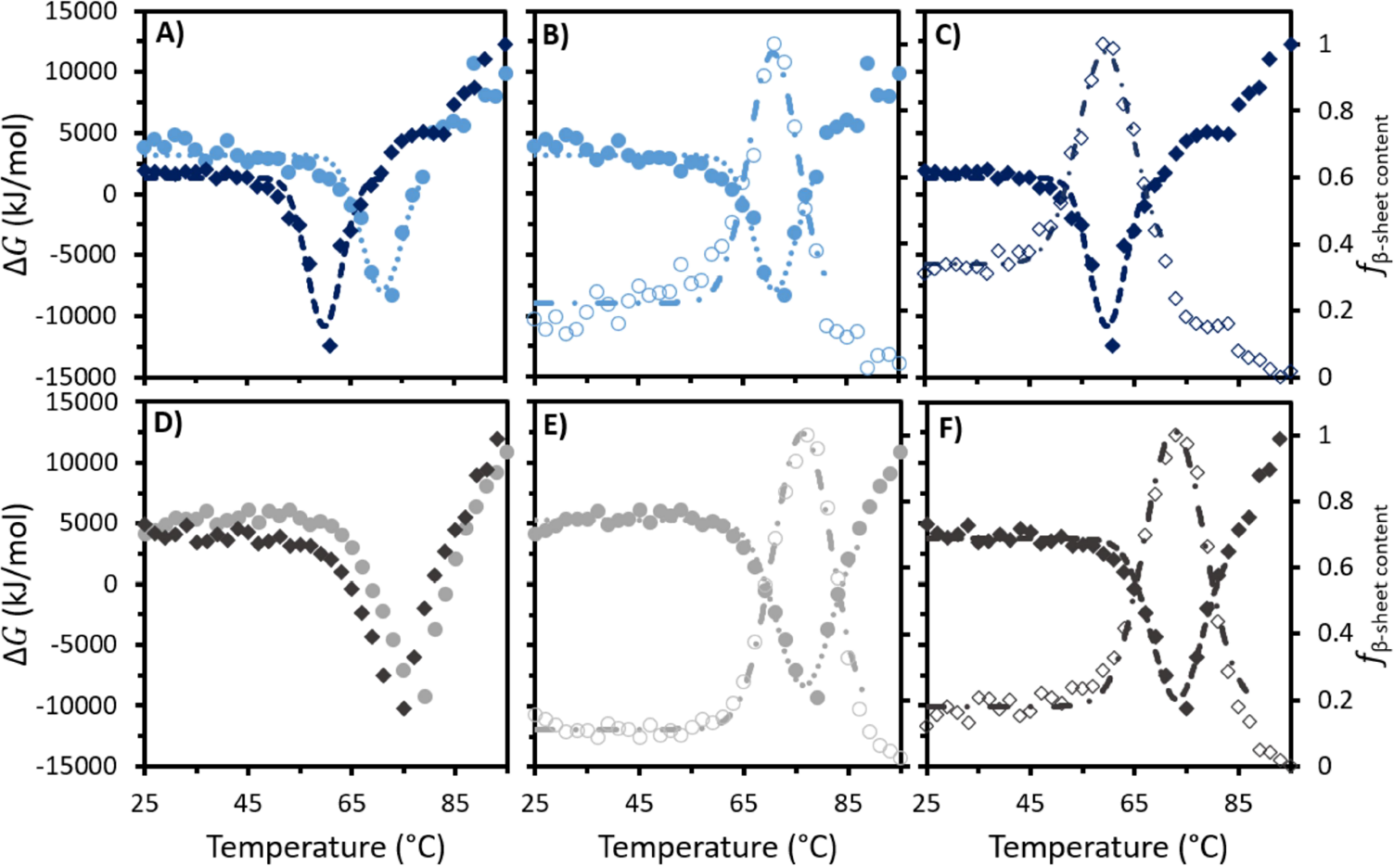
For IgG4 in water, overlays of (A) Δ*G*_f_ and Δ*G*_s_, (B) Δ*G*_f_ and *f*_f_β – sheet content__, and (C) Δ*G*_s_ and *f*_s_β – sheet content__. For IgG4 in 50 wt % [Cho]Cl, overlays of (D) Δ*G*_f_ and Δ*G*_s_, (E) Δ*G*_f_ and *f*_f_β – sheet content__, and (F) Δ*G*_s_ and *f*_s_β – sheet content__. Circles and diamonds represent the behavior of freshly prepared and stored samples, respectively, with dotted and dashed lines showing the Gaussian curves fitted to the data sets and used to calculate Δ*G*_min_. Solid symbols (left axis) and unfilled symbols (right axis) show Δ*G*_min_ and *f*_β – sheet content_, respectively. For all samples, see Figure S2.

To highlight the predictive power of our IL-based strategy, using the initially determined trends and calculated thermodynamics of fresh IgG4 systems to determine the behavior of stored systems, we overlayed Δ*G*_f_ and Δ*G*_s_, Δ*G*_f_ and *f*_f_β – sheet content__, andΔ*G*_s_ and *f*_s_β – sheet content__ (Figures 4 and S2). Evidently, for fresh and stored IgG4 in water (Figure 4A, B and C, respectively) and the equivalent three plots for fresh and stored IgG4 in 50 wt % [Cho]Cl (Figure 4D, E and F, respectively), we can clearly observe the distortion from the Gaussian model above 81 °C for water, also found above 83 °C for 10 wt % (Figure S1). We can see this is absent for 50 and 30 wt % (Figure S2), which gave a Gaussian distribution across the entire temperature range examined. Overall, our findings indicate that stored IgG4 in 50 wt % exhibits the highest thermodynamic stability, followed by 30 wt %, lowest for 10 wt % [Cho]Cl and water, as predicted.

### Determining and Validating Predictions of IgG4 Aggregation Propensity and Thermodynamic Properties

By additional thermodynamic calculations of each system, we further develop our strategy for predicting the thermodynamic properties and protein aggregation in solution over 365 days of storage at 4 °C. Initially, we found that the first melting transition of fresh IgG4 (*T*_f_m1__) in water (63.2 ± 0.8 °C) was lower than that for fresh [Cho]Cl solutions (69.8 ± 0.2, 67.8 ± 0.2, and 69.2 ± 0.1 °C for 10, 30, and 50 wt %, respectively) (Table 1). This indicated that in the absence of [Cho]Cl, IgG4 adopts conformations of lower thermal stability. In the fresh [Cho]Cl samples, higher concentrations served to reduce the initial change in entropy (Δ*S*_f_1__) (1370 ± 70 J/K/mol for 10 wt %, 1090 ± 50 J/K/mol for 30 wt %, and 1080 ± 20 J/K/mol for 50 wt %) and change in enthalpy (Δ*H*_f_1__) (96 ± 5 kJ/mol for 10 wt %, 74 ± 3 kJ/mol for 30 wt %, and 75 ± 1 kJ/mol for 50 wt %). However, both Δ*H*_f_2__ and Δ*S*_f_2__, associated with the second sigmoidal transition of the fresh samples, were raised with increasing [Cho]Cl concentrations (84 ± 5 kJ/mol and 1110 ± 60 J/K/mol, 108 ± 7 kJ/mol and 1280 ± 90 J/K/mol, and 137 ± 5 kJ/mol and 1650 ± 60 J/K/mol for 10, 30, and 50 wt % [Cho]Cl, respectively). Notably, the relatively higher Δ*H* and Δ*S* values for [Cho]Cl systems compared to solely water is logical, as the addition of [Cho]Cl molecules would lead to some structural rearrangement.^14,15^ Additionally, we observed that *T*_f_m2__, associated with the second sigmoidal transition of fresh samples, was comparable for water (77.7 ± 0.3 °C) and 10 wt % (76.6 ± 0.4 °C), yet raised for 30 wt % (84.3 ± 0.2 °C) and 50 wt % (82.9 ± 0.1 °C), indicating greater thermal stability of IgG4 in higher [Cho]Cl concentrations.

**Table 1:** *f*_β – sheet content_, Melting Temperature (*T*_m_), Enthalpy (Δ*H*), and Entropy (Δ*S*) for Each Sigmoidal Region of Fresh and Stored Samples of IgG4 in Water and Solutions of 10, 30, and 50 wt % [Cho]Cl, Heated from 25 to 95 °C. Determined from CD Data, As Described Previously^14^

sample	*T*_m1_ (°C)	Δ*H*_1_ (kJ/mol)	Δ*S*_1_ (J/K/ mol)	*T*_m2_ (°C)	Δ*H*_2_ (kJ/ mol)	Δ*S*_2_ (J/K/mol)	*T*_m3_ (°C)	Δ*H*_3_ (kJ/mol)	Δ*S*_3_ (J/K/mol)
water	63.2 ± 0.8	34 ± 2	530 ± 40	77.7 ± 0.3	100 ± 10	1300 ± 100			
water stored	52.8 ± 0.8	43 ± 4	810 ± 80	67.2 ± 0.2	62 ± 3	920 ± 50	85.8 ± 0.5	50 ± 10	600 ± 100
10%	69.8 ± 0.2	96 ± 5	1370 ± 70	76.6 ± 0.4	84 ± 5	1110 ± 60			
10% stored	69.5 ± 0.1	120 ± 3	1720 ± 50	75.8 ± 0.3	120 ± 10	1600 ± 200	92.2 ± 0.4	100 ± 20	1100 ± 200
30%	67.8 ± 0.1	74 ± 3	1090 ± 50	84.3 ± 0.2	108 ± 7	1280 ± 90			
30% stored	68.2 ± 0.1	81 ± 1	1180 ± 20	83.0 ± 0.1	145 ± 5	1740 ± 60			
50%	69.2 ± 0.1	75 ± 1	1080 ± 20	82.9 ± 0.1	137 ± 5	1650 ± 60			
50% stored	66.1 ± 0.1	75 ± 1	1130 ± 20	80.4 ± 0.2	96 ± 5	1200 ± 70			

Following 365 days of storage under 4 °C, all [Cho]Cl systems showed negligible changes in *T*_s_m1__ and *T*_s_m2__ (Table 1), yet for water we observe a significant decrease in both values (*T*_s_m1__ = 52.8 ± 0.8 °C and *T*_s_m2__ = 67.2 ± 0.2 °C). This emphasizes the reduced thermal stability as compared with [Cho]Cl solutions. Additionally, Δ*H*_s_1__ increased in all cases following storage, except for 50 wt % (75 ± 1 kJ/mol) that remained unchanged, and the value of Δ*S*_s_1__ showed smaller increases for 30 and 50 wt % (1180 ± 20 and 1130 ± 20 J/K/mol) compared to 10 wt % and water (810 ± 80 and 1720 ± 50 J/K/mol). Additionally, we observed a reduction in Δ*H*_s_2__ and Δ*S*_s_2__ for stored 50 wt % (96 ± 5 kJ/mol and 1200 ± 70 J/K/mol), yet an increase for 10 and 30 wt % (120 ± 10 kJ/mol and 1600 ± 200 J/K/mol for 10 wt % and 145 ± 5 kJ/mol and 1740 ± 60 J/K/mol for 30 wt %). Moreover, we found the expected third sigmoidal transition of stored water and 10 wt % with Δ*H*_s_3__ and Δ*S*_s_3__ (50 ± 10 kJ/mol and 600 ± 100 J/K/mol for water and 100 ± 20 kJ/mol and 1100 ± 200 J/K/mol for 10 wt %). Overall, these findings further demonstrate the enhanced thermal, structural, and thermodynamic stability of IgG4 with [Cho]Cl addition.

### MD Simulations for Assisting in Evaluating Predictions and Interpreting IgG4 Dynamics

**Figure 5:**
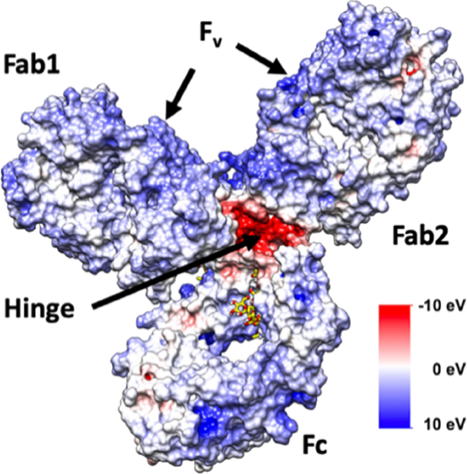
Electrostatic surface potential map for the IgG4 antibody with identifiable Fc, Fab, and hinge regions, with positive F_v_ regions shown on each Fab fragment, coloring of negative (red) to positive (blue) charge.

To gain further insight into our observed reduction in aggregation propensity and increase in IgG4 mean zeta potential at higher [Cho]Cl concentrations, we characterized IgG4 surface charge by mapping the electrostatic surface potential (ESP) of the IgG4 crystal structure (Figure 5). This mapping was carried out on the antibody protonation state +22 e at pH 5. It should be noted that while pH 5 is below the isoelectric point of IgG4,^28,39,40^ it was selected because the calculated protonation states of IgG4 were found to remain constant in the pH range between approximately 4.5 and 5.5.

We found the majority of positive charge to be located on the variable region (F_v_) of the Fab fragments (Fab1 and Fab2) of the investigated IgG4. A lower number of positively charged regions were located on the C_γ_2–C_γ_3 interface of the Fc fragment, known to bind via different motifs that vary depending on the protein interactions.^41^ We also observed that the exposed negatively charged regions on the antibody surface are predominantly localized on the IgG4 hinge, with smaller negatively charged regions found on the Fc and Fab fragments. On the basis of this, it is expected that the large number of positive regions found on the IgG4 ESP map leads to the preferential exclusion of [Cho]Cl species in solution, and extensive electrostatic interactions and hydrogen bonding between the [Cho]Cl molecules and IgG4 chain restrict IgG4 aggregation at higher concentrations.^42^

As we developed our predictive IL-based strategy, we carried out MD simulations at 25 °C, starting from the crystal structure of Pembrolizumab (PDB ID – 5DK3)^43^ solvated in water and in 10, 30, and 50 wt % [Cho]Cl of increasing ionic strength. We also performed simulations at 97 °C to examine the dynamics of IgG4 and correlate our findings with the experimentally determined thermodynamic stability of IgG4 at elevated temperatures.

In this work, we computed preferential interaction coefficients, Γ_23,_ by means of minimum distance distribution functions (MDDFs) and the Kirkwood–Buff (KB) theory of solvation. This approach has previously been used to examine aqueous IL-protein interactions.^14,44^ Additionally, we employed the indistinguishable ion approach to account for the cationic and anionic co-solute species in the calculation of Γ_23_, whereby the preferential interaction coefficients of the choline cation and the chloride anion were calculated separately and then combined to provide a single Γ_23_ for the co-solutes. This method has been recently shown to provide reasonable results for ionic excipients^45^ and ILs.^46^

From our simulations at 25 °C, we found that the Γ_23_ values decreased with increasing [Cho]Cl concentration, from −1.0 ± 0.12 to −2.8 ± 0.18 and −3.1 ± 0.15 for 10, 30, and 50 wt %, respectively. This indicated that [Cho]Cl is preferentially excluded from the antibody surface for each system and acts as a protectant, thus stabilizing the protein^38,39^ and reducing IgG4 aggregation propensity, most significantly at 50 wt %, in agreement with our experimental results. These findings are consistent with previous work showing the protectant effect of [Cho][DHP] on cytochrome C,^47^ ribonuclease A,^25^ and human serum albumin,^48^ resulting in enhanced stabilization of these systems.

Next, we examined the minimum distance distribution functions (MDDFs), providing insight into the individual ion-IgG4 interactions and details of the probability of finding the choline and chloride ions relative to the antibody surface (Figure S3). We found that for each [Cho]Cl system examined, overall, the choline cations preferentially interact with the antibody surface as opposed to the chloride anions. However, while at low concentrations the weakly hydrated chloride anions are distributed throughout the solution, the occurrence of chloride anions interacting with the IgG4 surface increases with [Cho]Cl concentration. This is indicated by the absence of a distinct short-range peak, typically observed in the MDDFs. Specifically, from hydrogen bonding analysis, we determined that the choline cations can act as both hydrogen bond donors and acceptors. For example, we found that the hydroxyl groups of the choline cations form hydrogen bonds with the oxygen atoms of the asparagine groups, including ASN141, ASN214, ASN533, and ASN876, on IgG4. Additionally, amino groups on the indole ring of the tryptophan groups, prominently TRP814, TRP990, and TRP1041, on IgG4 formed hydrogen bonds with the oxygen atoms of the choline cations. We also found that the choline cations interact via hydrogen bonds with the cystine amino acids, such as CYX218, on the IgG4 hinge region. Furthermore, our results are in line with previous studies of specific ion effects on protein-IL interactions in aqueous ILs,^49−51^ which have found that large organic cations preferentially interact directly with the antibody surface, whereas the behavior of the anions is highly dependent on the basicity of the anion and the strength of the anion–water interactions.^52−56^

We consider that IgG antibodies are highly flexible due to the composition of the hinge region, which allows for a relatively unhindered motion of the two Fab arms in solution.^41,57,58^ Accounting for this, we examined the global structure of IgG4 in water and each [Cho]Cl solution, allowing us to assess the effect of ionic strength on IgG4 dynamics and correlate this to our experimental results. This was achieved by calculating the root mean squared deviation (RMSD) (Figure S4) and assessing the Fab-Fab and Fab-Fc distances calculated between the center-of-mass of each domain (Figure S5). Additionally, we examined the root mean squared fluctuations (RMSF) to further understand the effect of ionic strength, varied by [Cho]Cl concentration, on the dynamics of the individual amino acids in the IgG4 chain.

At 25 °C, RMSD values of the individual Fab and Fc fragments (Figure S4) revealed that the individual IgG4 domains remained relatively stable. Moreover, we found that the increased RMSD for the Fab1 and Fc fragments in water and 10 wt % [Cho]Cl solutions correspond to the formation of a Fab-Fc interface. This was confirmed by investigation of the Fab-Fab and Fab-Fc distances (Figure S5). Notably, the change in RMSD and corresponding formation of a Fab-Fc interface was not observed for IgG4 in 30 and 50 wt % [Cho]Cl. From the RMSF we find that, in general, the fluctuations of the individual amino acids are dampened with increasing ionic strength. This is consistent with previous protein-IL simulation studies that have shown that the addition of ILs reduces protein dynamics.^59^

**Figure 6:**
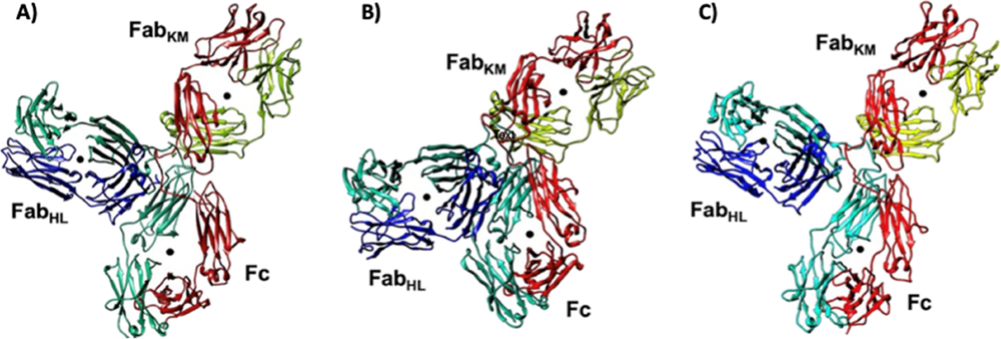
(A) Y-shaped structure from the crystal structure of IgG4 (PDB ID-5DK3);^43^ (B) λ-shaped structure found to dominate in water and 10 wt % at 25 °C; (C) Y-shaped structure as found for the 50 wt % [Cho]Cl simulations at both 25 and 97 °C.

We note that the X-ray crystal structure (Figure 6A) presents an idealized Y-shaped conformer of the antibody. Upon simulating in water and in 10 wt %, we found that IgG4 transitioned to adopting a λ-shaped conformer (Figure 6B). For the 30 and 50 wt % [Cho]Cl solutions, we observed that a Y-shaped conformer was preferred over the λ–shaped conformer of IgG4 (Figure 6C). Based on previous MD simulations, a λ-shaped conformer has been found for the anti-natriuretic peptide receptor A (NPRA) IgG4 antibody.^58^ Accordingly, in our simulations, the λ-shaped conformer arises via the formation of an Fc-Fab interface (predominantly Fc–Fab_HL_). Notably, we did not observe the equally probable formation of the Fc–Fab_KM_ interface, possibly due to the unique rotation of the Fc CH_2_ region associated with the Pembrolizumab IgG4 which may block Fc-Fab association.

In line with our experimental results and predictions, by the 97 °C simulations we found a general increase in the antibody fragment motions, particularly the Fab arms and Fc tail, in the case of water and 10 wt % [Cho]Cl. This is indicated by the greater RMSD (Figure S6) values and the preferential formation of a Fab-Fc interface and the λ-shaped conformer.^58^ Specifically, we observed enhanced flexibility and motion of the Fab fragments, compared to the higher concentration [Cho]Cl solutions. Additionally, at 97 °C, we observed a greater relative orientation and movement of one of the Fab arms and Fc tail of the antibody in 30 wt % [Cho]Cl (Figures S6 and S7), thereby facilitating a transition from the Y-shaped conformer to the λ-shaped conformer. Notably, this is in contrast to our observation of the Y-shaped conformer preferred over the λ–shaped conformer of IgG4 for 30 wt % at 25 °C. For 50 wt %, we observed a preference for the Y-shaped conformer at both 25 and 97 °C. This indicates that the increased [Cho]Cl concentration restricts the formation of the λ–shaped conformer. We note that the transition from the Y-shaped conformer to the λ-shaped conformer cannot be entirely discounted as longer simulations may reveal this transition; however, this is unlikely based on our thermodynamic calculations.

## Discussion

In deciphering the effects of [Cho]Cl on IgG4 aggregation propensity, we considered that the ability of aqueous [Cho]Cl-based ILs to stabilize, denature, or induce the aggregation of proteins is, to some extent, comparable to conventional co-solutes, such as guanidinium chloride, triethylamine-*N*-oxide, and urea.^16,52^ In particular, a detailed theoretical framework for the application of KB theory and preferential binding models have been presented for examining the interactions between ILs and proteins, such as the interaction of aqueous alkylimidazolium- based ILs with peptides and proteins.^44,60^ Based on our experimental data, thermodynamic calculations, and observations from the MD simulations, we suggest that the charge screening effect of [Cho]Cl, IgG4 compaction, and the formation of a strong network of [Cho]Cl molecules around IgG4 provide the stabilization and reduced aggregation propensity at higher [Cho]Cl concentrations. Specifically, we propose that the choline cations form hydrogen bonds and hydrophobic interactions with the charged amino acids and protein backbone, stabilizing the folded conformations of IgG4.^14^ Together, the chloride anions can contribute to enhanced structural stability against collapse by electrostatic interactions,^14,15^ forming a viscoelastic interfacial stabilization network hindering changes in the protein structure, adsorption at the air–water interface, and increase in dynamic surface tension.^16,61−63^ Furthermore, the observed Y-shaped conformer adopted in 30 and 50 wt % at 25 °C suggests dampening of the dynamics for these systems, possibly due to preferential interactions of IgG4 with [Cho]Cl in concentrated solutions. From this, we infer that water is excluded from the vicinity of the protein surface, also supported by the highest Γ_23_ values observed for water. Accordingly, for water and 10 wt % [Cho]Cl, the formation of the Fab-Fc interface and increase in IgG4 conformational flexibility can be explained by the unavailability of sufficient [Cho]Cl molecules for compaction via electrostatic and hydrogen bonding interactions at the surface, leading to the enhanced aggregation propensity.

Given that fresh 30 and 50 wt % showed more negative Δ*G*_f_min__ values, we suggest that the [Cho]Cl molecules bind to IgG4 to stabilize the initial conformation, having cooperative folding transitions with a strong bias toward native interactions.^14,35,64,65^ As such, adding [Cho]Cl can raise the free energy barrier of unfolding, limiting intermediate states, and thus hindering aggregation.^14,35,37,64−66^ In line with the higher folding energy barriers promoting the transition of misfolded forms into the folded conformations,^35,64,65^ the less negative value of Δ*G*_f_min__ for 10 wt % and the third sigmoidal region found for water suggest that the IgG4 conformational space is explored so that high energy regions are avoided. Consequently, the conversions between the folded and misfolded states are more frequent,^35,37,64^ explaining the greater aggregation propensity observed for water and 10 wt %. Additionally, the relatively higher *T*_f_m1__ and *T*_f_m2__ for the [Cho]Cl systems compared to water corroborate the claim that with [Cho]Cl addition, we can expect conformations of greater thermal stability and folded states not accessible in water. We propose that for each system, IgG4 flexibility should be finely balanced with thermal stability. To illustrate, we observed a decrease in Δ*H*_f_1__ and Δ*S*_f_1__ with increasing [Cho]Cl concentration, reflecting that the entropic contribution to IgG4 thermal stability is much smaller for fresh 30 and 50 wt % compared to 10 wt % [Cho]Cl, consistent with our previous work.^14,15^ This suggests that the thermal stability is coupled to the enhanced conformational stability of the folded state.^64−67^ Following this logic, fresh IgG4 in 10 wt % [Cho]Cl showed the highest Δ*H*_f_1__ and Δ*S*_f_1__ values, demonstrating that the enhanced thermal stability of this system is a consequence of an increase in the conformational entropy of the folded state ensembles. Furthermore, Δ*H*_f_2__ and Δ*S*_f_2__ were the lowest for 10 wt % and comparable for fresh water and 30 wt % [Cho]Cl, with the [Cho]Cl systems showing significantly higher amounts of secondary structure. This indicates a tendency to adopt a more native-like folded conformation at *T*_f_m2__ for IgG4 in [Cho]Cl. Thus, we suggest that if stabilizing interactions are perturbed, increasing the entropy of the folded state could lead to stabilization, provided these exceed the loss of enthalpic contribution.^14,15,35,64^

Following 365 days of storage under 4 °C, our DLS, zeta potential, and CD measurements demonstrated that IgG4 in water is highly prone to aggregation and the addition of [Cho]Cl served to preserve native residue–residue interactions over storage. This was highlighted by the observations of greatest loss in secondary structure, largest change in Δ*G*_s_min__, and lowest *T*_s_m1__ and *T*_s_m2__ all in water. Based on our initial results, for IgG4 in 50 wt %, we expected conformational changes to be suppressed due to confinement effects.^15,35−38^ In this case, we predicted that stored 50 wt %, with most native contacts, would show an increase in Δ*H*_s_1__ and Δ*S*_s_1__ and decrease in Δ*S*_s_2__ and Δ*H*_s_2__, relative to the fresh systems. We found that for the [Cho]Cl systems examined, only stored 50 wt % showed a decrease in Δ*H*_s_2__and Δ*S*_s_2__ and the lowest Δ*S*_s_2__. This aligns with our initial predictions and proposal that charge screening, compaction, and long-range hydrophobic contacts at higher [Cho]Cl concentrations can restrict IgG4 aggregation.^14,15,64−67^ Accordingly, for stored 30 wt %, which showed an increase in Δ*H*_s_1__, Δ*S*_s_1__, Δ*H*_s_2__, and Δ*S*_s_2__ and a more negative Δ*G*_s_min__ compared to 10 wt %, the enhanced thermal and structural stability observed can be attributed to compaction and greater solution viscosity. This is also supported by the greater RMSD value found for water and 10 wt % [Cho]Cl at the 97 °C MD simulations, and the preferential formation of a Fab-Fc interface and greater motion of the Fab fragments compared to 30 and 50 wt %. Moreover, in line with our initial predictions, we observed a third subtle transition, *T*_s_m3__, for stored 10 wt % [Cho]Cl and water, of greatest aggregation propensity. We rationalize that conformational changes and bond formations between the amino acid residues can increase the entropic cost, and the ability of IgG4 to adopt multiple conformations can lead to an increase in surface hydrophobicity and aggregation propensity.^64−67^ Notably, as IgG4 in water is expected to potentially populate a large number of conformations for a given type of fold,^7,14,64^ we can obtain conformationally stable yet misfolded β-sheet-rich structures of low thermal stability, explaining the more negative Δ*G*_s_min__ of stored water. We suggest that in higher [Cho]Cl concentrations, a small number of conformations are consistent with forming a thermodynamically stable antibody, and we can construct systems in which tight packing between [Cho]Cl and the amino acid side chains leads to enhanced IgG4 thermal, structural, and thermodynamic stabilization over extended storage. Notably, there is a growing demand for novel pharmaceutical excipients, with multifaceted and unique properties, to aid in suppressing aggregation and prolonging the shelf life of protein therapeutics.^1−3,13^ However, in order to circumvent the requirements of compiling extensive supportive information and regulatory approval, the pharmaceutical sector remains heavily reliant on well-established and pre-approved excipients.^2,3,5^ Introducing ILs into the pharmaceutical development pipeline has immense potential for enhancing the quality and performance of protein therapeutic candidates and opening new avenues for formulation innovation.

## Conclusions

We developed an IL-based strategy for predicting the thermodynamic stability and aggregation propensity of IgG4, intrinsically prone to aggregation in solution. We examined the stresses of pH, ionic strength, and temperature and evaluated the structural, thermal, and thermodynamic stability and aggregation propensity of IgG4 in water and 10, 30, and 50 wt % [Cho]Cl. We developed our predictive approach in two stages. First, we conducted DLS, zeta potential, and variable temperature CD experiments to systematically assess each fresh system. Based on initial results, we predicted the aggregation propensity and thermodynamic properties of IgG4 in each system following 365 days under 4 °C. Upon re-evaluation of each system following extended storage, we found reduced aggregation propensity with increasing [Cho]Cl concentration, highest for water. We propose that for the solutions of higher ionic strength and pH levels below the isoelectric point of IgG4, the charge screening effect and preferential binding of the [Cho]Cl molecules to the IgG4 surfaces enable compaction and enhanced thermodynamic stability. This is supported by our experimental findings and thermodynamic calculations, as well as our MD simulations showing reduction of IgG4 motion at higher [Cho]Cl concentrations. We demonstrated that by adding [Cho]Cl, it is possible to guide conformational sampling via [Cho]Cl-local residue interactions that bias IgG4 chain fragments toward forming secondary structures of reduced aggregation propensity. This likely involves favorable long-range interactions, local packing, side chain hydrogen bonding, and burial of hydrophobic amino acids away from water. Thus, our IL-based approach for predicting protein aggregation propensity and thermodynamic stability can be thought of in the framework of reversing a protein-folding problem. Instead of searching for the lowest energy conformation for a given protein, the goal is to determine the solution conditions that will stabilize a desired conformation or binding interaction. Based on the validation of our thermodynamically controlled predictions, we achieved a high level of success. We envision that our strategy can be applied to a wide range of [Cho]-based ILs and deep eutectic solvents for predicting and enhancing the thermodynamic and storage stability of diverse proteins with novel folds.

## Materials and Methods

### Materials and Formulation Preparation

IgG4 was produced as previously described.^14^ [Cho]Cl was purchased from Sigma-Aldrich Company Limited (Gillingham, Dorset, UK) and stored as recommended. Ultrapure water was used for preparing the formulations and obtained from a PURELAB Ultra water purifier (ELGA LabWater, High Wycombe, UK) with resistivity of 18.2 MΩ. An aqueous stock solution of [Cho]Cl was prepared by addition of ultrapure water to the salt, then shaking at room temperature for 1 hour to give 80 wt % [Cho]Cl solution. From the 80 wt % solution, aqueous stock solutions of 10, 30, and 50 wt % [Cho]Cl were prepared. Each aqueous stock [Cho]Cl solution contained 20 mg mL^–1^ of IgG4, filtered using 0.22 μm syringe filter units (Thermo Fisher Scientific Inc., Waltham, MA USA). All formulations were immediately stored at 4 °C until measured. Prior to a measurement, a sample was prepared from each stock IgG4-[Cho]Cl solution, with IgG4 filtered and diluted to a concentration of 0.4 mg/mL. For the experimental measurements performed, these conditions were previously found optimum.^14−16^ For each solution, IgG4 concentration was confirmed by measurements on the NanoDrop One ThermoFischer Scientific Inc. (Thermo Fisher Scientific Inc., Waltham, MA USA).

### pH DLS and Zeta Potential Measurements

The pH of each solution was measured using the pH electrode Mettler Toledo InLab Micro (WOLFLABS, Pocklington, York, UK). As previously described,^16^ to investigate the change in *D*_h_ and PDI, DLS measurements were performed using a Zetasizer Nano ZS (Malvern Panalytical Ltd., Malvern, UK) at a 90° scattering angle; and to determine the surface charge, zeta potential measurements were performed using the Litesizer 500 (Anton Paar GmbH, Ostfildern, Germany). For each experiment, the average of three results is reported. All samples were measured under identical conditions, fresh and following 365 days of storage at 4 °C.

### Variable Temperature CD Spectroscopy Measurements and Analysis

To examine the structural, thermal, and thermodynamic stability of the formulations, temperature variable CD experiments were performed on a Chirascan CD spectrometer (Applied Photophysics Ltd., Leatherhead, Surrey, UK) in combination with a Quantum Northwest Peltier temperature controller (Quantum Northwest Inc., Liberty Lake, WA, USA), as previously described.^14,15^ Samples were measured with a heating rate of 1 °C/minute between 25 and 97 °C at 2 °C intervals between 200 and 260 nm. Samples were measured fresh and following 365 days of storage at 4 °C. CD data was processed and analyzed following the methodology described previously.^14^

Briefly, Origin software (OriginLab Corporation, Northampton, MA, USA) was used to zero and smooth the data. MRE was calculated, and the data at 218 nm was converted into a plot of the relative fraction of the β-sheet content, *f*_β – sheet content_, of the protein at each temperature, calculated by logistic regression as follows:
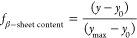
1where *y* is the experimentally measured CD signal, *y*_0_ is the minimum magnitude CD signal, and *y*_max_ is the strongest CD signal (maximum of the negative peak), with *f*_β – sheet content_ = 0 at *y* = *y*_0_ and *f*_β – sheet content_ = 1 at *y* = *y*_max_.

A Gaussian curve was fitted to each data set with the peak width and area under the curve extrapolated directly from this fit. Percentage change in the area and width between fresh and stored samples were calculated, relative to the fresh sample, with a positive change indicating an increase (and vice versa) (Table S3).

The thermodynamics parameters of each transition were calculated using the two-stage model, fitted to a sigmoidal curve. The fraction of protein in the final conformation (*f*) was then calculated as shown in eq 1, and the equilibrium constant was determined as
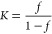
2and from there, the free energy of the transition, Δ*G*_m_, was calculated as
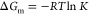
3for ideal gas constant *R*. Δ*G*_m_ was plotted linearly against *T* in the transition region (−5 kJ mol^–1^ < Δ*G*_m_ < 5 kJ mol^–1^), with *T*_m_ as the midpoint of the sigmoid and the point at which exactly half of the molecules are the initial and final ensembles and Δ*G*_m_ = 0. The enthalpy (Δ*H*_m_) and entropy (Δ*S*_m_) of each transition were estimated from the Δ*G*_m_ and *T*_m_ using^14^
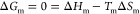
4Δ*G*_m_ was also plotted against *T* across the entire temperature range examined, resulting in a curve which was fitted to an inverted Gaussian, the minimum of which was defined as Δ*G*_min_.

### MD Simulations and Analysis

The structure of the IgG4 antibody was modeled based on the crystal structure of the full length IgG4 antibody, Pembrolizumab (resolution 3.2 Å, PDB entry: 5DK3).^43^ The original crystal structure consisted of an IgG4 dimer, from which we employed only the A (light) and B (heavy) chains and retained the glycan chains in our simulations. Missing residues were generated using Modeller prior to assigning protonation states. The protonation state of the antibody was assigned at pH 5.0 using the H++ webserver.^68^ This produced an overall charge of +22 e for the antibody. To account for potential local changes in the protonation state at the lower pH of IgG4 in the [Cho]Cl systems, protonation states were also determined at pH 4.5 and 5.5. At these pH values, the protonation state was found to remain at +22 e.

For aqueous simulations, one IgG4 was solvated in a cubic TIP3P water box with a minimal distance of 14 Å between the boundaries of the box and the nearest protein atoms using the tleap module in Ambertools18.^69^ For simulations containing [Cho]Cl, the number of [Cho]Cl and water species was calculated to retain the same box size as the aqueous simulations while maintaining the experimental concentrations. Table S6 shows the number of [Cho]Cl and water molecules used to simulate IgG4 in 10, 30, and 50 wt % [Cho]Cl. Each simulation cell contained one full antibody molecule. This generated simulation boxes containing ∼490 to 570 k atoms. The starting structures for each these systems was generated using Packmol.^70^ The corresponding disulfide and glycan linkages and parameter and topology files were generated using tleap.

Protein residues were simulated using the Amber ff14SB force field.^71^ Amber consistent parameters were employed for the chloride anions,^72^ while choline cations were described using the General Amber Force Field (GAFF)^73^ and RESP charges.^74^ Subsequently, charges on the species were scaled to 0.8. This led to an imbalance of charge in each system of −2 e, which was then balanced with the addition of 2 Na^+^ ions.^72^ This approach toward neutralizing the overall system charge has previously been shown to have little effect on the protein structure and dynamics.

All solvated structures were then subjected to two-stage energy minimization. In the first stage of 5000 steps, the protein was restrained to its crystallographic positions using a harmonic potential with a force constant of 25 kcal/(mol Å^2^) while all other atoms were unrestrained. In the second stage of 5000 steps, no restraints were applied. In both stages, the steepest descent method was used for the 1st 2500 steps and the conjugate gradient method was switched on for the rest of the steps. Each system was then subjected to 500 ps of heating (NVT) and 1 ns pre-equilibration in the NPT ensemble to obtain the required temperature and density. Three independent 150 ns simulations at 25 °C and two independent 300 ns simulations at 97 °C were performed for each system in the NPT ensemble. Notably, high temperature simulations were carried out to improve the dynamics of the highly viscous systems and further examine the validity of the IL-based predictive approach and conclusions obtained from the experimental analysis and thermodynamic calculations. The higher temperature of 97 °C was selected based on previous work, which revealed that an IgG Fab fragment retains its secondary structure in IL-water solutions at 127 °C, with no denaturation observed.^14^ All simulations were carried out using Amber18,^69^ employing the same settings as used in previous work.^14^ Primary analysis of each trajectory was carried out with CPPTRAJ.^75^

To probe the role of [Cho]Cl in enhancing the stability or the aggregation propensity of IgG4, we computed the preferential interaction coefficients of each system. This was performed using the ComplexMixtures (v0.4.13) software with *R* = 12 Å^44^ and was carried out using the last 50 ns of each of the simulations at 25 °C. In brief, the preferential interaction coefficient, Γ_23_, of an antibody with an excipient can be calculated by applying eq 5, where the subscripts 1, 2, and 3 refer to water, protein, and excipient, respectively.^76,77^
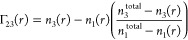
5

To account for the ionic species, the choline cations and chloride anions, we employed the indistinguishable ion approach, shown to be a reasonable approximation for ILs.^46,78^ In this approach, Γ_23_ values are calculated separately for each species, then combined using eq 6.^79^
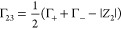
6

In this equation, Γ_+_ and Γ_–_ refer to the preferential interaction coefficients for the cation and anion, respectively, and Z_2_ is the net charge of the protein. We performed separate simulations constraining the Na^+^ ions at distances >10 Å from the protein surface. Then, the preferential interaction coefficients were computed, which revealed that the inclusion of the two Na^+^ ions had a negligible effect on the calculated properties.

## Supporting Information Available

For each sample, pH, DLS, and zeta potential measurement values and statistical analysis; CD data analysis includes MRE values and percentage change analysis; width and area of Gaussian calculations; Δ(Δ*G*_min_) calculations; zoomed in plots of *f*_β – sheet content_ and Δ*G*; *G*_min_ and *f*_β – sheet content_ overlaid plots for each system; MD simulations analysis includes the number of molecules used to simulate each system; details of fragment motions and RMSD analysis and calculations; plots of representative MDDFs and calculated contributions; representative RMSD plots of individual IgG4 domains at 25 and 97 °C; and plots showing the differences and distances between individual IgG4 fragments at 25 and 97 °C for each system (PDF)
