# Supplementary material for: Ionic Liquid-Based Strategy for Predicting Protein Aggregation Propensity and Thermodynamic Stability
Source: JACS Au. 2022 Sep 9;2(9):2068–80. doi: 10.1021/jacsau.2c00356 (PMC9516703; doi:10.1021/jacsau.2c00356)
Supplement: Supplementary file 1 — au2c00356_si_001.pdf [file au2c00356_si_001.pdf]

Supporting Information for

Ionic Liquid-Based Strategy for Predicting Protein Aggregation Propensity and Thermodynamic Stability

Talia A. Shmool,[a] Laura K. Martin,[b] Richard P. Matthews,[a] Jason P. Hallett*[a]

[a]Department of Chemical Engineering, Imperial College London, South Kensington Campus, London SW7 2AZ, UK
E-mail: j.hallett@imperial.ac.uk
Tel: +44 (0)20 7594 5388

[b]Department of Engineering Science, University of Oxford, Parks Road, Oxford OX1 3PJ, UK

List of figures and tables

- For each sample, pH, DLS and zeta potential measurement values, and statistical analysis of DLS and zeta potential data.
- CD data analysis includes MRE values and percentage change analysis, width and area of Gaussian calculations, $\Delta(\Delta G_{\mathrm{min}})$ calculations, zoomed in plots of the $f_{\mathrm{\beta-sheet\ content}}$ and ΔG, and G_{min} and $f_{\mathrm{\beta-sheet\ content}}$ overlaid plots for each system.
- MD simulations analysis includes number of molecules used to simulate each system, details of fragment motions and RMSD analysis and calculations, plots of representative MDDFs and the calculated contributions, representative RMSD plots of individual IgG4 domains at 25 and 97 °C, and plots showing the differences and distances between individual IgG4 fragments at 25 and 97 °C for each system.

Table S1. pH, D_h, PDI and mean zeta potential values of IgG4 in water and solutions of 10, 30, and 50 wt% [Cho]Cl, measured fresh and after storage for 365 days at 4 °C.
Table S2. Minimum MRE values for fresh and stored samples at 218 nm under each condition, and the percentage change between the initial and final values.
Table S3. Calculated width and area of the Gaussian curves fitted to the $f_{\beta-sheet\ content}$ plots for IgG4 in water and 10, 30, and 50 wt% [Cho]Cl solutions.
Table S4. Residual sum of squares values for the Gaussian fits to $f_{\beta-sheet\ content}$ data for the fresh and stored samples for 365 days at 4 °C.
Table S5. $\Delta(\Delta G_{\mathrm{min}})$ for IgG4 in water, 10, 30 and 50 wt% [Cho]Cl.
Figure S1. Zoomed in plots of the $f_{\mathrm{\beta-sheet\ content}}$ and ΔG for IgG4 in water and 10 wt% [Cho]Cl, shown to highlight the deviations from the Gaussian models at high temperatures. Pale blue (water) and red (10 wt%) circles show the fresh data, while dark blue (water) and red (10 wt%) diamonds show the samples after 365 days of storage at 4 °C.
Figure S2. ΔG_{min} (filled circle left axis) and $f_{\mathrm{\beta-sheet\ content}}$ (unfilled symbols, right axis) overlaid for each IgG4 condition.
Table S6. Number of [Cho]Cl and water molecules used to simulate each system. Each simulation cell contains one IgG4 antibody.
Figure S3. Representative minimum distance distribution functions (MDDFs) for the constituent solvent species A) choline, B) chloride and C) water interacting with the antibody surface at 10, 30 and 50 wt% [Cho]Cl. We have also included the calculated contributions attributed to the various classes of amino acids, including charged (navy), polar (red), and hydrophobic (light blue).
Figure S4. Representative RMSD plots of the individual Fab1 (red), Fab2 (black) and Fc (blue) domains for IgG4 in each system at 25 °C.
Figure S5. A) Relationship between the Fab-Fab distance (d1) and the absolute difference in the Fab-Fc distances (abs(d2-d3)) from the IgG4 simulations for each system. B) Relationship between the Fab-Fab distance (d1) and the absolute difference in the Fab-Fc distances (abs(d2-d3)) from the IgG4 simulations for water and 10, 30, 50 wt% [Cho]Cl at 25 °C.
Figure S6. Representative RMSD plots of the individual Fab1 (red), Fab2 (black) and Fc (blue) domains for IgG4 in each system at 97 °C.
Figure S7. Relationship between the Fab-Fab distance (d1) and the absolute difference in the Fab-Fc distances (abs(d2-d3)) from the IgG4 simulations for water, 10, 30, and 50 wt% [Cho]Cl at 97 °C.

pH, dynamic light scattering and zeta potential measurements

Table S1. pH, hydrodynamic diameter (**D_h**), PDI and mean zeta potential values of IgG4 in water and solutions of 10, 30, and 50 wt% [Cho]Cl, measured fresh and after storage of 365 days at 4 °C.

Sample	pH Fresh	pH Stored	D_h Fresh (nm)	D_h Stored (nm)	PDI Fresh	PDI Stored	Mean zeta potential Fresh (meV)	Mean zeta potential Stored (meV)
Water	6.35	6	410 ± 10	649 ± 8	0.7 ± 0.3	0.7 ± 0.3	2.3 ± 0.8	-1.4 ± 0.8
10%	5.86	5.79	307 ± 8	460 ± 20	0.6 ± 0.1	0.6 ± 0.1	6.2 ± 0.6	4.5 ± 0.7
30%	5.73	5.65	201 ± 8	274 ± 8	0.6 ± 0.2	0.6 ± 0.1	6.6 ± 0.5	5.7 ± 1.1
50%	5.47	5.05	135 ± 4	159 ± 1	0.56 ± 0.06	0.6 ± 0.2	9.5 ± 0.9	8.5 ± 1.1

Statistical analysis of DLS and zeta potential data

A two-way ANOVA was performed. The significance of the effect of [Cho]Cl concentration and the effect of long-term storage on the D_h and zeta potential values for each system were determined. Data was grouped into fresh and stored IgG4 samples, and the distinct [Cho]Cl concentrations were considered as different treatment conditions within each group.

In both fresh and stored groups, when concentrations of [Cho]Cl were compared pairwise, all pairs of D_h values were statistically significantly different at the 0.0001 significance level. When the effect of long-term storage was examined, D_h values for fresh versus stored samples were different at the 0.0001 significance level for IgG4 in water, 10 wt% and 30 wt% [Cho]Cl. For IgG4 in 50 wt% [Cho]Cl the difference between fresh and stored samples was only significant at the 0.05 level. Overall, both [Cho]Cl concentration variation and storage were found to have significant effects on D_h values at the 0.0001 level.

For the zeta potential data obtained for IgG4, when [Cho]Cl concentrations were compared pairwise, mean zeta potential values were significantly different at the 0.01 level or below, with the exception of 10 and 30 wt% [Cho]Cl for which the difference in means was not significant. This behaviour was the same for both the fresh and stored sample groups. When fresh and stored samples were compared, only IgG4 in water gave a significant change in zeta potential value to the 0.001 level. For all [Cho]Cl systems this change was not significant. Overall, the value of zeta potential for IgG4 was found to be affected by both [Cho]Cl concentration and storage to the 0.0001 significance level.

Circular dichroism spectroscopy

Table S2. Minimum mean residual ellipticity (MRE) values for fresh and stored samples at 218 nm under each condition, and the percentage change between the initial and final values.

Sample	Initial MRE_f (deg cm^2 $dmol^{-1}$)	Min MRE_f (deg cm^2 $dmol^{-1}$)	ΔMRE_f (%)	Initial MRE_s (deg cm^2 $dmol^{-1}$)	Min MRE_s (deg cm^2 $dmol^{-1}$)	ΔMRE_s (%)	ΔMRE_i with storage (%)	ΔMRE_{min} with storage (%)
Water	-12,800	-16,700	30	-14,400	-18,800	30	13	13
10%	-3,200	-6,200	95	-7,200	-10,800	49	126	73
30%	-2,800	-7,300	164	-6,000	-11,000	84	115	50
50%	-3,200	-7,000	119	-3,000	-6,600	116	5	6

Width and area of Gaussian calculations

To further evaluate the data, a Gaussian curve was fitted to the $f_{\beta-sheet\ content}$ plots with the width at half peak height measured and the area under the curve integrated for each system. When comparing the peak width and area under the curve, Table S2 shows that IgG4 in 30 and 50 wt% [Cho]Cl exhibited wider peaks and larger integrated areas in both fresh and stored samples compared to water and 10 wt% [Cho]Cl. Following storage, 50 wt% [Cho]Cl showed the smallest relative change in both peak width and area.

Table S3. Calculated width and area of the Gaussian curves fitted to the $f_{\beta-sheet\ content}$ plots for IgG4 in water and 10, 30, and 50 wt% [Cho]Cl solutions.

Sample	Area Fresh	Area Stored	Area % change	Width Fresh	Width Stored	Width % change
Water	8.9 ± 0.6	8.5 ± 0.4	- 4.7	9.4 ± 0.6	10.6 ± 0.4	12.5
10%	4.3 ± 0.2	3.9 ± 0.2	-9.4	5.1 ± 0.3	5.8 ± 0.3	14.1
30%	17.1 ± 0.4	12.3 ± 0.5	-28.2	16.8 ± 0.4	13.6 ± 0.5	-18.4
50%	12.6 ± 0.4	12.0 ± 0.5	-4.3	13.3 ± 0.4	13.6 ± 0.5	2.4

Table S4. Residual sum of squares (RSS) values for the Gaussian fits to $f_{\beta-sheet\ content}$ data for the fresh and stored samples for 365 days at 4 °C.

Sample	RSS Fresh	RSS Stored
Water	0.11259	0.02353
10%	0.04007	0.03089
30%	0.03247	0.05272
50%	0.07623	0.03038

Table S5. $\Delta(\Delta G_{\mathrm{min}})$ for IgG4 in water, 10, 30 and 50 wt% [Cho]Cl.

Sample	RSS ΔG_{f} fit	RSS ΔG_{s} fit	$\Delta G_{\mathrm{f_{min}}}$ (kJ/mol)	$\Delta G_{s_{\mathrm{min}}}$ (kJ/mol)	$\Delta(\Delta G_{\mathrm{min}})$ kJ/mol)
Water	2.38E07	2.21E07	-8 ± 1	-11 ± 1	-3 ± 2
10%	2.03E06	1.61E06	-3.3 ± 0.4	-4.6 ± 0.4	-1.3 ± 0.6
30%	5.06E07	7.85E06	-7 ± 1	-7.9 ± 0.6	-1 ± 1
50%	1.85E07	1.61E07	-8.3 ± 0.9	-9.5 ± 0.9	-1 ± 1

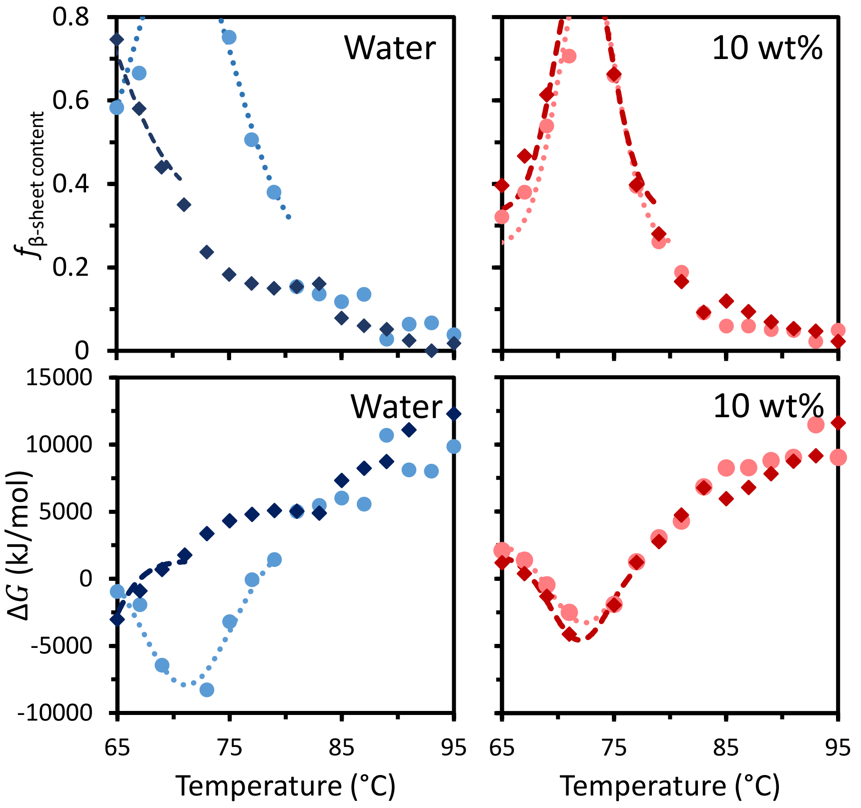

Figure S1. Zoomed in plots of the $f_{\beta-\text{sheet content}}$ and ΔG for IgG4 in water and 10 wt% [Cho]Cl, shown to highlight the deviations from the Gaussian models at high temperatures. Pale blue (water) and pale red (10 wt%) circles show the fresh data, while dark blue (water) and dark red (10 wt%) diamonds show the samples after 365 days of storage at 4 °C.

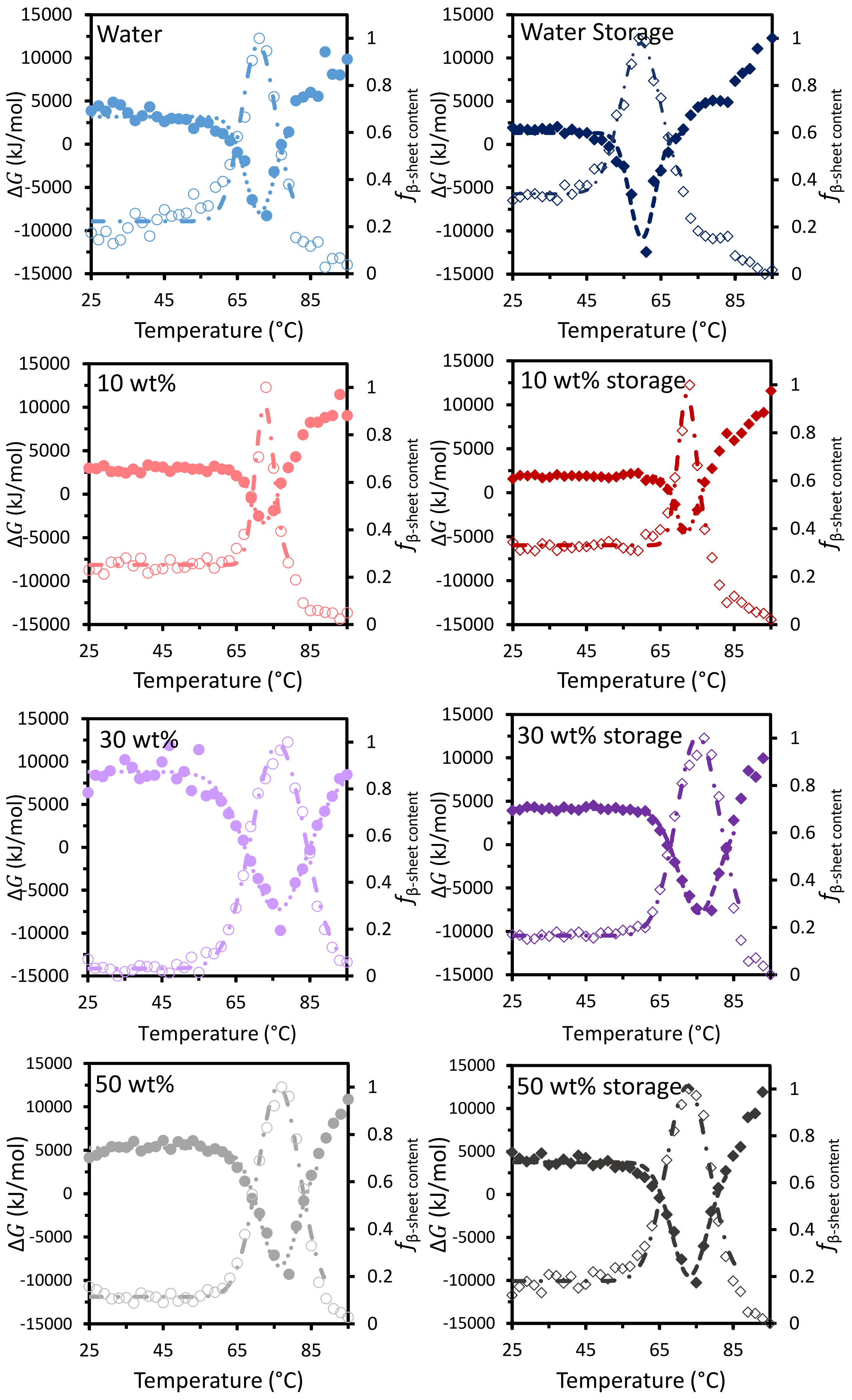

Water
Water Storage
10 wt%
10 wt% storage
30 wt%
30 wt% storage
50 wt%
50 wt% storage
ΔG (kJ/mol)
$f_{\beta\text{-sheet content}}$
Temperature (°C)
15000
10000
5000
0
-5000
-10000
-15000
1
0.8
0.6
0.4
0.2
0
25
45
65
85

Figure S2. ΔG_{min} (filled circle left axis) and $f_{\beta-\mathrm{sheet\ content}}$ (unfilled symbols, right axis) overlaid for each IgG4 condition (water, 10, 30 and 50 wt% [Cho]Cl]).

Molecular dynamics simulations

The number of [Cho]Cl and water molecules used to simulate the 10, 30 and 50 wt % [Cho]Cl systems are provided in Table S6. Each simulation cell contained one full IgG4 antibody and thirty additional free chloride anions, to ensure the overall charge of each system was balanced.

Table S6. Number of [Cho]Cl and water molecules used to simulate each system. Each simulation cell contains one IgG4 antibody.

[Cho]Cl solution (wt%)	Number of [Cho]Cl ion pairs	Number of waters
10	1978	140000
30	6812	130000
50	11428	100000

Root Mean Squared Deviation – Domains of IgG4

The root mean squared deviation (RMSD) of backbone atoms, using the crystal structure as a reference, was mass-weighted and averaged over each frame. To obtain domain RMSD values, only root-mean-square fitting and RMSD calculations for the domains of interest (Fab1, Fab2 and Fc) were determined. In this work, the domains were defined as follows: For Fab1, residues 1−225 of heavy chain 1 and residues 1−218 of light chain 1; Fab2, residues 1−225 of heavy chain 2 and residues 1−218 of light chain 2; and Fc, residues 240−447 of heavy chain 1 and heavy chain 2; and hinge, residues 240−447 of heavy chain 1 and heavy chain 2.

RMSD calculations for the individual Fab and Fc fragments (Figures S4 and S6) revealed that the domains remained relatively stable during the simulations. This stability was greatly enhanced on increasing the [Cho]Cl concentration as evidenced by the smaller RMSD for the 50 wt% [Cho]Cl systems. Moreover, the increase in RMSD observed for the Fab1 and Fc fragments in the water, 10 and 30 wt% [Cho]Cl in the region 150-300 ns, corresponds to the formation of the observed Fab-Fc interface.

Fragment Motions

The motions of the IgG4 fragments influence the degree of antigen binding by allowing the fragments to adopt a variety of orientations. Due to the stability observed within the fragments we applied a set of distance criteria previously employed[1] to investigate the IgG4 conformers adopted during our MD simulations.

In this approach, we first defined the distances between the centre of masses of the individual Fab and Fc fragments. These were defined as d1 (Fab1-Fab2), d2 (Fab1-Fc) and d3 (Fab2-Fc) (Figure S5A). To gain insight into potential Fab-Fab and Fab-Fc interactions we then plotted d1 against the absolute difference between d2 and d3, abs(d2-d3), for IgG4 in each system (Figure S5B and S7). This plot presents a simple indicator of the conformational preference and symmetry of IgG4 by examining the relation between Fab-Fab distance (d1) and the absolute difference in the Fab-Fc distances (abs(d2-d3)). The corresponding scatter plots revealed a conformational preference for λ–shaped conformer for water, 10 and 30 wt% solutions as indicated by the larger d1 (70-80 Å) and abs(d2-d3) (10-30 Å). The 50 wt% solution revealed a preference for a Y-shaped conformer, with smaller d1 (55-70 Å) and abs(d2-d3) (0-20 Å) values.

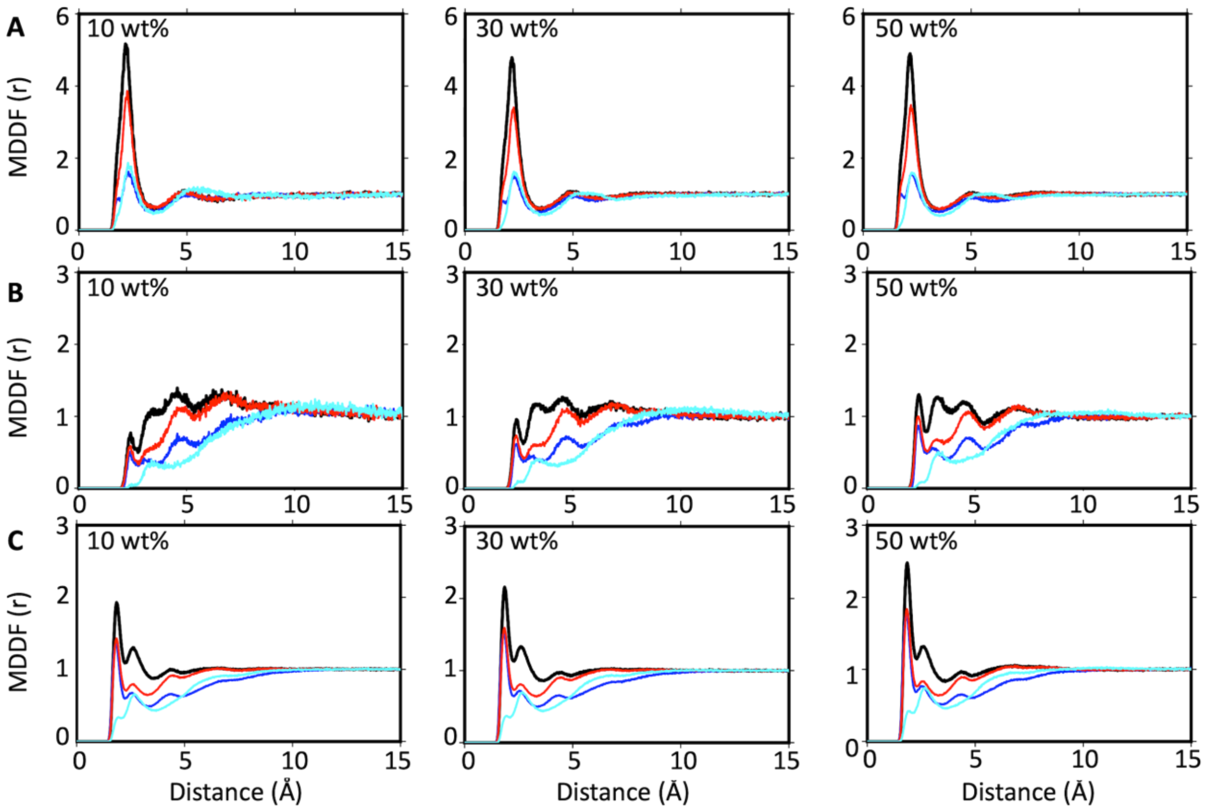

Figure S3. Representative minimum distance distribution functions (MDDFs) for the constituent solvent species A) choline, B) chloride and C) water interacting with the antibody surface at 10, 30 and 50 wt% [Cho]Cl. Also included, the calculated contributions attributed to the various classes of amino acids, including charged (navy), polar (red), and hydrophobic (light blue).

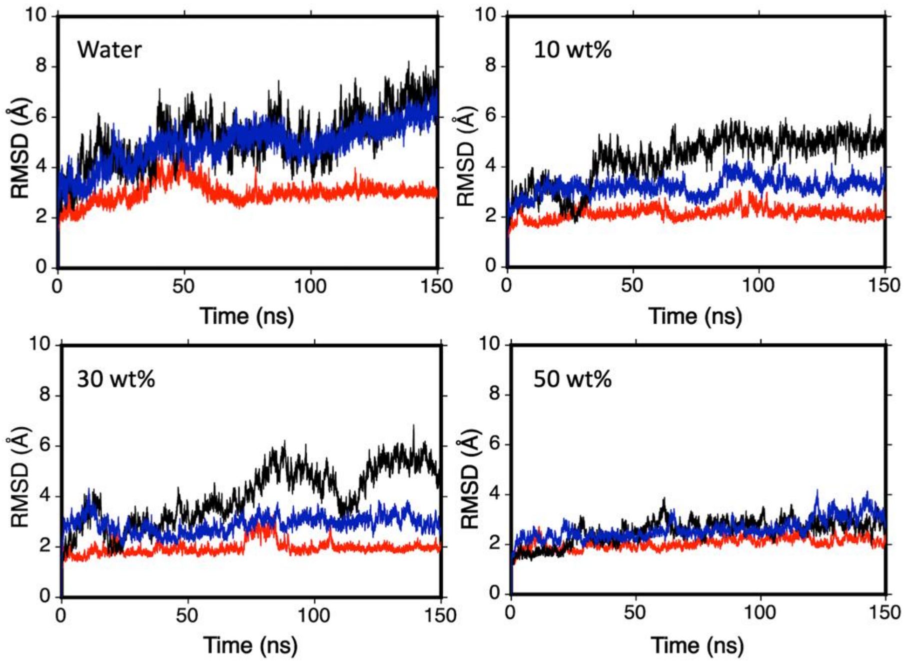

Figure S4. Representative RMSD plots of the individual Fab1 (red), Fab2 (black) and Fc (blue) domains for IgG4 in each system at 25 °C.

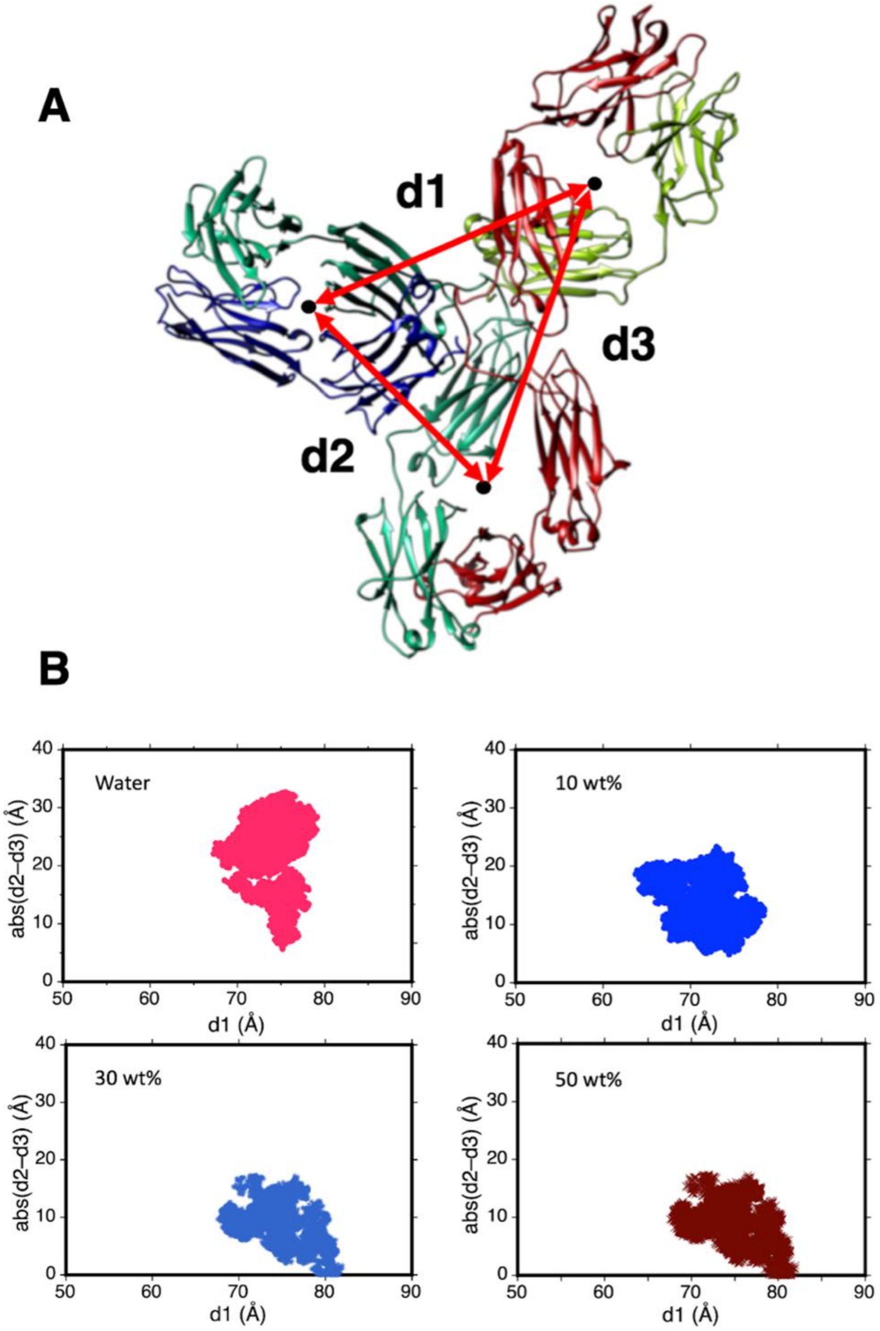

Figure S5. A) Relationship between the Fab-Fab distance (d1) and the absolute difference in the Fab-Fc distances (abs(d2-d3)) from the IgG4 simulations for each system. B) Relationship between the Fab-Fab distance (d1) and the absolute difference in the Fab-Fc distances (abs(d2-d3)) from the IgG4 simulations for water and 10, 30, 50 wt% [Cho]Cl at 25 °C.

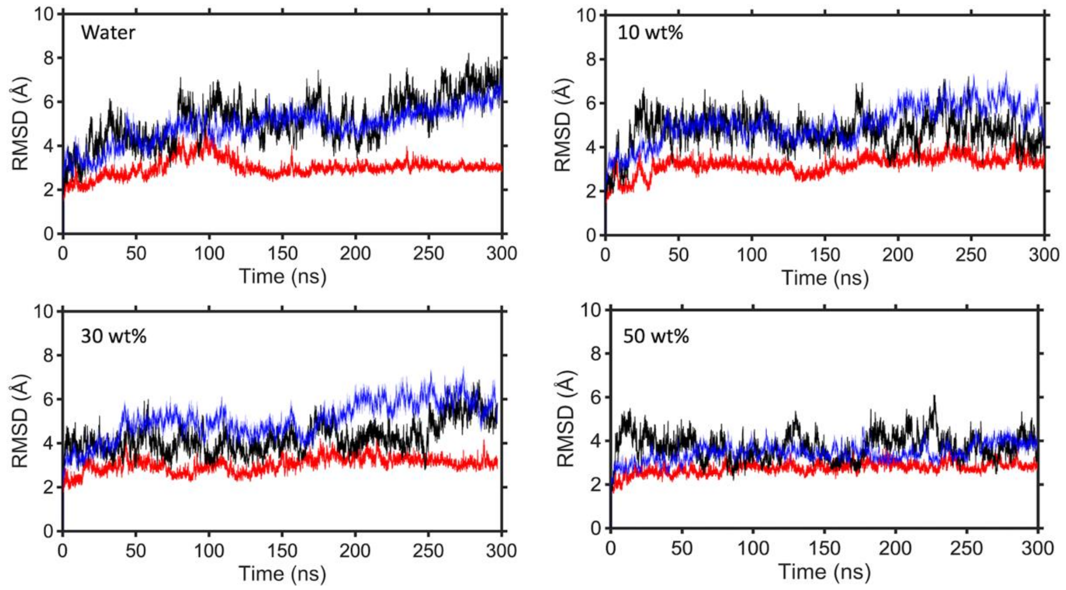

Figure S6. Representative RMSD plots of the individual Fab1 (red), Fab2 (black) and Fc (blue) domains for IgG4 in each system at 97 °C.

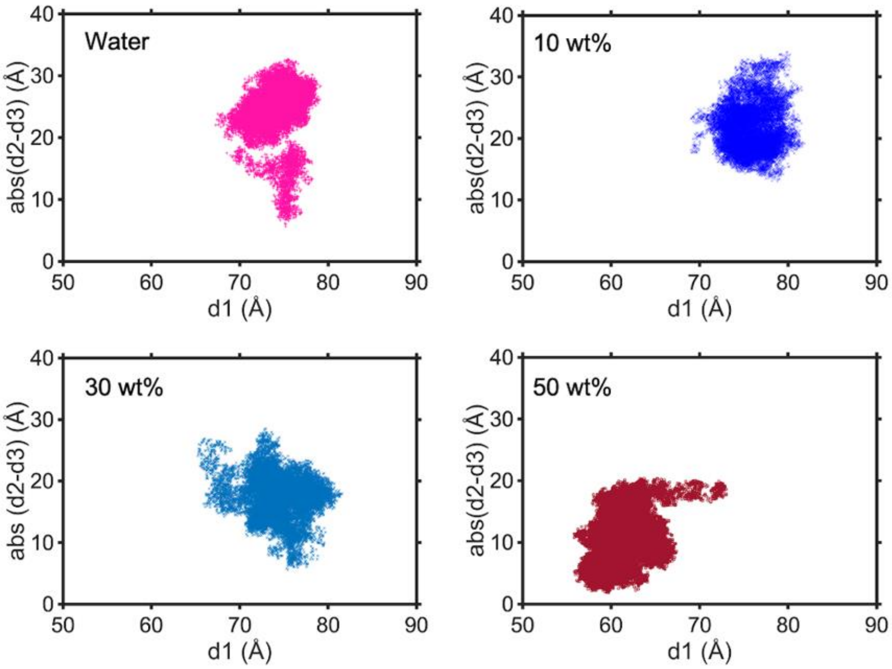

Figure S7. Relationship between the Fab-Fab distance (d1) and the absolute difference in the Fab-Fc distances (abs(d2-d3)) from the IgG4 simulations for water, 10, 30, and 50 wt% [Cho]Cl at 97 °C.

References

(1) Wright, D. W.; Elliston, E. L. K.; Hui, G. K.; Perkins, S. J. Atomistic Modeling of Scattering Curves for Human IgG1/4 Reveals New Structure-Function Insights. *Biophys. J.* **2019**, *117* (11), 2101–2119. https://doi.org/10.1016/j.bpj.2019.10.024.
